# Unraveling the electrophilic oxygen-mediated mechanism for alcohol electrooxidation on NiO

**DOI:** 10.1093/nsr/nwad099

**Published:** 2023-04-13

**Authors:** Wei Chen, Jianqiao Shi, Chao Xie, Wang Zhou, Leitao Xu, Yingying Li, Yandong Wu, Binbin Wu, Yu-Cheng Huang, Bo Zhou, Ming Yang, Jilei Liu, Chung-Li Dong, Tehua Wang, Yuqin Zou, Shuangyin Wang

**Affiliations:** State Key Laboratory of Chemo/Bio-Sensing and Chemometrics, College of Chemistry and Chemical Engineering, Advanced Catalytic Engineering Research Center of the Ministry of Education, Hunan University, Changsha 410082; State Key Laboratory of Chemo/Bio-Sensing and Chemometrics, College of Chemistry and Chemical Engineering, Advanced Catalytic Engineering Research Center of the Ministry of Education, Hunan University, Changsha 410082; State Key Laboratory of Chemo/Bio-Sensing and Chemometrics, College of Chemistry and Chemical Engineering, Advanced Catalytic Engineering Research Center of the Ministry of Education, Hunan University, Changsha 410082; College of Chemistry and Chemical Engineering, Hunan Normal University, Changsha 410081; College of Materials Science and Engineering, Hunan University, Changsha 410082; State Key Laboratory of Chemo/Bio-Sensing and Chemometrics, College of Chemistry and Chemical Engineering, Advanced Catalytic Engineering Research Center of the Ministry of Education, Hunan University, Changsha 410082; State Key Laboratory of Chemo/Bio-Sensing and Chemometrics, College of Chemistry and Chemical Engineering, Advanced Catalytic Engineering Research Center of the Ministry of Education, Hunan University, Changsha 410082; State Key Laboratory of Chemo/Bio-Sensing and Chemometrics, College of Chemistry and Chemical Engineering, Advanced Catalytic Engineering Research Center of the Ministry of Education, Hunan University, Changsha 410082; State Key Laboratory of Chemo/Bio-Sensing and Chemometrics, College of Chemistry and Chemical Engineering, Advanced Catalytic Engineering Research Center of the Ministry of Education, Hunan University, Changsha 410082; Research Center for X-ray Science & Department of Physics, Tamkang University, New Taipei City 25137; State Key Laboratory of Chemo/Bio-Sensing and Chemometrics, College of Chemistry and Chemical Engineering, Advanced Catalytic Engineering Research Center of the Ministry of Education, Hunan University, Changsha 410082; State Key Laboratory of Chemo/Bio-Sensing and Chemometrics, College of Chemistry and Chemical Engineering, Advanced Catalytic Engineering Research Center of the Ministry of Education, Hunan University, Changsha 410082; College of Materials Science and Engineering, Hunan University, Changsha 410082; Research Center for X-ray Science & Department of Physics, Tamkang University, New Taipei City 25137; State Key Laboratory of Chemo/Bio-Sensing and Chemometrics, College of Chemistry and Chemical Engineering, Advanced Catalytic Engineering Research Center of the Ministry of Education, Hunan University, Changsha 410082; State Key Laboratory of Chemo/Bio-Sensing and Chemometrics, College of Chemistry and Chemical Engineering, Advanced Catalytic Engineering Research Center of the Ministry of Education, Hunan University, Changsha 410082; Shenzhen Institute of Hunan University, Shenzhen 518057; State Key Laboratory of Chemo/Bio-Sensing and Chemometrics, College of Chemistry and Chemical Engineering, Advanced Catalytic Engineering Research Center of the Ministry of Education, Hunan University, Changsha 410082; Shenzhen Institute of Hunan University, Shenzhen 518057

**Keywords:** nucleophile oxidation reaction, alcohol electrooxidation, organic electrosynthesis, nickel-based electrocatalysts, C–C bond cleavage

## Abstract

Aqueous organic electrosynthesis such as nucleophile oxidation reaction (NOR) is an economical and green approach. However, its development has been hindered by the inadequate understanding of the synergy between the electrochemical and non-electrochemical steps. In this study, we unravel the NOR mechanism for the primary alcohol/vicinal diol electrooxidation on NiO. Thereinto, the electrochemical step is the generation of Ni^3+^-(OH)_ads_, and the spontaneous reaction between Ni^3+^-(OH)_ads_ and nucleophiles is an electrocatalyst-induced non-electrochemical step. We identify that two electrophilic oxygen-mediated mechanisms (EOMs), EOM involving hydrogen atom transfer (HAT) and EOM involving C–C bond cleavage, play pivotal roles in the electrooxidation of primary alcohol to carboxylic acid and the electrooxidation of vicinal diol to carboxylic acid and formic acid, respectively. Based on these findings, we establish a unified NOR mechanism for alcohol electrooxidation and deepen the understanding of the synergy between the electrochemical and non-electrochemical steps during NOR, which can guide the sustainable electrochemical synthesis of organic chemicals.

## Teaser

This work unravels the electrophilic oxygen-mediated mechanism for alcohol electrooxidation on NiO, and elaborates the synergy between the electrochemical and non-electrochemical steps for the alcohol electrooxidation on NiO.

## INTRODUCTION

Under the escalating severity of environmental and energy-related concerns, organic electrosynthesis in aqueous electrolytes, such as electrooxidation/electroreduction of organic compounds, electrochemical CO_2_ reduction reaction (eCO_2_RR), *etc.*, has started to garner increasing attention as a sustainable and green approach [1–3]. Aqueous organic electrosynthesis differs from the traditional aqueous electrochemical reactions, e.g. hydrogen/oxygen evolution reaction (HER/OER), because it may involve both the electrochemical step and non-electrochemical process involving organic compounds [4,5]. However, electrochemists sometimes excessively emphasize the roles of the electrocatalyst and electrochemical step, ignoring the non-electrochemical process. This severely hinders the development of aqueous organic electrosynthesis. Recently, numerous aqueous organic electrosynthesis reactions, which sounded almost impossible to achieve previously, have been realized by regulating spontaneous non-electrochemical processes (such as coupling and rearrangement) [6–9]. Researchers have gradually realized the importance of the synergy between the electrochemical and non-electrochemical steps so as to develop new organic electrosynthesis reactions. Nucleophile (such as alcohols, aldehydes, amines, *etc.*) oxidation reaction (NOR) based on nickel-based electrocatalysts is an essential part of aqueous organic electrosynthesis [2,10–14]. Nevertheless, almost all existing NOR systems were proposed decades ago, suggesting a lack of breakthroughs in this area [10–14]. Establishing a unified NOR mechanism is critical for developing new NOR systems, and the key lies in comprehensively understanding the synergy between electrochemical and non-electrochemical steps.

Notably, the nucleophile oxidation pathway is almost the same across different nickel-based electrocatalysts [2,10–15]. However, the oxidation pathways of different nucleophiles may differ for the same nickel-based electrocatalyst [16,17]. To research the NOR mechanism, consider alcohol electrooxidation for instance. On nickel-based electrocatalysts, primary alcohol (R-CH_2_OH) can be electrochemically oxidized to carboxylic acid (R-COOH) [2,10]. Vicinal diol (R-CHOH-CH_2_OH) can be electrochemically oxidized to R-COOH and formic acid (HCOOH) accompanied by the C–C bond cleavage [16–18]. It remains unknown as to why the R-CH_2_OH and R-CHOH-CH_2_OH electrooxidation pathways differ significantly. As such, to gain an insight into the NOR mechanism, it is necessary to address the following three key issues: (1) What are the electrochemical and non-electrochemical steps in NOR? (2) How do the electrochemical/non-electrochemical steps collaborate during NOR? (3) How does the synergy between electrochemical and non-electrochemical steps determine the nucleophile electrooxidation pathway?

Here, we explore alcohol electrooxidation on nickel oxide (NiO). The electrochemical step is directly related to the electrocatalyst. We identify two types of non-electrochemical steps, i.e. (1) electrocatalyst-induced non-electrochemical step and (2) electrocatalyst-irrelevant non-electrochemical step. Alcohol electrooxidation on NiO is an indirect electrooxidation reaction with Ni^3+^-(OH)_ads_ containing electrophilic oxygen as the redox mediator, and it follows the electrophilic oxygen-mediated mechanism (EOM), which comprises the electrochemical step (i.e. electrochemical generation of Ni^3+^-(OH)_ads_) and electrocatalyst-induced non-electrochemical step (e.g. Ni^3+^-(OH)_ads_–induced hydrogen atom transfer and Ni^3+^-(OH)_ads_–induced C–C bond cleavage). Consequently, there are two electrocatalyst functions for alcohol electrooxidation on NiO, i.e. (1) EOM involving hydrogen atom transfer (HAT) and (2) EOM involving C–C bond cleavage. Owing to the synergy of the EOM involving HAT and hydration reaction (which is an electrocatalyst-irrelevant non-electrochemical step), R-CH_2_OH can be electrochemically oxidized to R-COOH on NiO. Because of the synergy between the EOM involving HAT and EOM involving C–C bond cleavage, R-CHOH-CH_2_OH can be electrochemically oxidized to R-COOH and HCOOH on NiO. As such, we establish a unified NOR mechanism for alcohol electrooxidation, which can pave the way for developing new NOR systems and other organic electrosynthesis reactions.

## RESULTS AND DISCUSSION

### Electrophilic oxygen-mediated mechanism involving hydrogen atom transfer

The unique relevance between Ni^2+^/Ni^3+^ redox couple and NOR results in excellent NOR performance of nickel-based electrocatalysts, which have been extensively used in NOR, e.g. alcohol electrooxidation [2,10–18]. In this study, NiO was adopted as the model NOR electrocatalyst to study the electrocatalyst function for alcohol electrooxidation. NiO hexagonal nanosheets with a lateral size range of 40–60 nm were synthesized successfully; the detailed characterizations are shown in Figs S1–S5. A 1 M KOH solution and nucleophile-containing 1 M KOH were employed as the electrolytes for the OER and NOR systems, respectively. Under an oxidation potential, the anode will lose electrons to generate electron-deficient species, which may be able to attack the nucleophile as the active NOR intermediate [19,20]. The OER and NOR systems based on NiO were analyzed and compared to unravel the electrocatalyst function in the NOR system.

**Figure 1.**
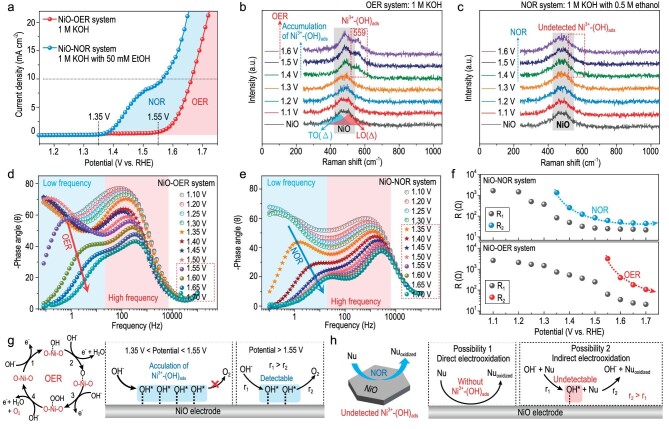
OER and NOR systems based on NiO. (a) Anodic polarization curves of NiO in the OER system (1 M KOH) and NOR system (1 M KOH with 50 mM ethanol). (b and c) *In situ* Raman spectra of NiO electrode in the OER system (b) and NOR system (1 M KOH with 0.5 M ethanol) (c). (d and e) Bode plots for the NiO electrode at different potentials in the OER system (d) and NOR system (1 M KOH with 0.5 M ethanol) (e). (f) Equivalent resistances (R_1_ and R_2_) versus potential for the NiO electrode in the OER and NOR systems. (g and h) Schematic diagrams illustrating the OER (g) and NOR (h) systems based on NiO.

The onset potentials of OER and NOR (the model NOR system: the ethanol electrooxidation) for NiO are ∼1.55 and ∼1.35 V, respectively (Fig. 1a). The Raman spectrum of fresh NiO electrode shows a broad peak at ∼485 cm^−1^, which is fitted by employing two components including two-phonon (2P) transverse optical (TO(Δ); peak at ∼460 cm^−1^) and 2P longitudinal optical (LO(Δ); peak at ∼505 cm^−1^) modes [21,22]. For the *in situ* Raman spectra of NiO in the OER system, a new peak at ∼559 cm^−1^, attributed to the stretching vibration of OH adsorption on Ni^3+^ site (Ni^3+^-(OH)_ads_), appears at a potential above 1.35 V, indicating the electrochemical generation of Ni^3+^-(OH)_ads_ (Figs 1b and S6) [23–26]. However, during the NOR on NiO at a potential above 1.35 V, the Ni^3+^-(OH)_ads_ intermediate cannot be detected by *in situ* Raman spectroscopy (Fig. 1c). Operando electrochemical impedance spectroscopy (EIS) was performed to analyze the reaction interface for the NiO electrode in the OER/NOR system (Figs S7–S10, Tables S1 and S2) [27–29]. According to analysis of the reaction interface, both OER and NOR occur at the low-frequency interface between the surface hydrous layer and electrical double layer (EDL) (Fig. 1d–f) [30–32]. Hence, the OER intermediates i.e. OH_ads_, O_ads_, and OOH_ads_, are generated at the low-frequency interface, and the NOR active intermediate may be one of the OER intermediates [19].

There are two processes for NiO in the OER system (Fig. 1g) [31]: (1) Between ∼1.35 to ∼1.55 V, the hydroxyl ion is electrochemically adsorbed to form Ni^3+^-(OH)_ads_, i.e. OH_ads_, but it cannot be further oxidized to oxygen, thereby resulting in the accumulation of Ni^3+^-(OH)_ads_ [19,27]; (2) At a potential above ∼1.55 V, Ni^3+^-(OH)_ads_ can be electrochemically oxidized to oxygen [32]. There are two possibilities for the undetectable Ni^3+^-(OH)_ads_ during NOR on NiO (Fig. 1h) [33]: (1) Nucleophiles can be directly electrochemically oxidized, without generating Ni^3+^-(OH)_ads_; (2) The NOR on NiO is an indirect electrooxidation with Ni^3+^-(OH)_ads_ as the redox mediator, and the rate of the reaction between Ni^3+^-(OH)_ads_ and nucleophiles is far larger than that of the electrochemical generation of Ni^3+^-(OH)_ads_, thereby yielding the undetectable Ni^3+^-(OH)_ads_.

**Figure 2.**
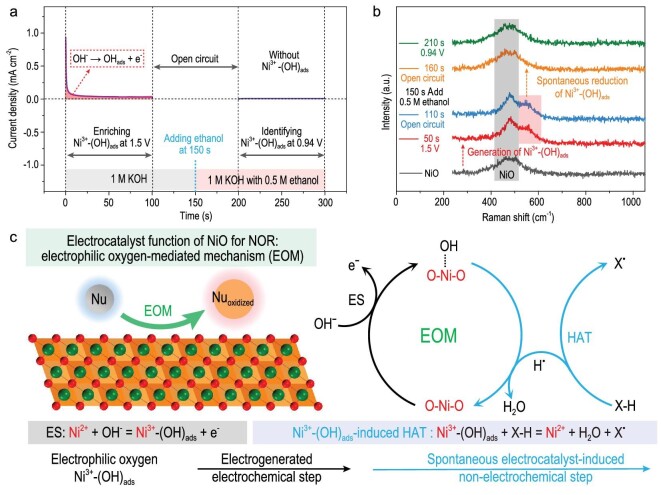
Electrocatalyst function for NOR on NiO. (a) Current density versus time for the NiO electrode (0 to 100 s: 1.5 V; 100 to 200 s: open-circuit condition; 200 to 300 s: 0.94 V) in different electrolytes (0 to 150 s: 1 M KOH; 150 to 300 s: 1 M KOH with 0.5 M ethanol). (b) Synchronous *in situ* Raman spectra for the NiO electrode in the electrochemical testing. (c) EOM involving HAT including the electrochemical generation of Ni^3+^-(OH)_ads_- and Ni^3+^-(OH)_ads_–induced HAT.

The Ni^3+^-(OH)_ads_ accumulates at a high potential (e.g. 1.50 V) in the OER system, and the electroreduction of Ni^3+^-(OH)_ads_ occurs at a low potential (e.g. 0.94 V) (Fig. S11) [34]. According to the multi-potential steps for the NiO electrode in the OER system and synchronous *in situ* Raman spectra, after accumulating Ni^3+^-(OH)_ads_ at 1.50 V, the generated Ni^3+^-(OH)_ads_ still existed in 1 M KOH under an open-circuit condition, and the accumulated Ni^3+^-(OH)_ads_ could be electrochemically reduced at 0.94 V (Fig. S12). To identify the role of Ni^3+^-(OH)_ads_ in NOR, the electrochemical generation of Ni^3+^-(OH)_ads_ should be separated from NOR. At 1.50 V in 1 M KOH (0 to 100 s), Ni^3+^-(OH)_ads_ was formed and accumulated; the generated Ni^3+^-(OH)_ads_ still existed in 1 M KOH under an open-circuit condition (100 to 150 s); after adding 0.5 M ethanol into 1 M KOH at 150 s, the accumulated Ni^3+^-(OH)_ads_ intermediates were consumed completely by ethanol under the open-circuit condition (Fig. 2a and b). Furthermore, the oxidation product for the reaction between Ni^3+^-(OH)_ads_ and ethanol is acetic acid (Fig. S13). These results prove that Ni^3+^-(OH)_ads_ can spontaneously catalyze the oxidative dehydrogenation of alcohol (e.g. ethanol) to carboxylic acid without an applied voltage.

The hydrogen atom transfer (HAT) reaction between Ni^3+^-(OH)_ads_ and nucleophiles (e.g. R-CH_2_OH) plays an important role in the nucleophile dehydrogenation oxidation pathway for NOR [10,35]. Although both the Ni^3+^ site and electron-deficient OH_ads_ can work as the electron acceptor, the proton acceptor can only be the electrophilic oxygen in Ni^3+^-(OH)_ads_, instead of the Ni^3+^ site [10,19]. The electrophilic oxygen in Ni^3+^-(OH)_ads_ can spontaneously grab the hydrogen atom from nucleophiles, and this electrocatalyst-induced non-electrochemical step is defined as Ni^3+^-(OH)_ads_–induced HAT (Fig. 2c). The NOR on NiO is a unique indirect electrooxidation reaction with the Ni^3+^-(OH)_ads_ containing electrophilic oxygen as the redox mediator [3,10]. Hence, in the NOR system, NiO follows the electrophilic oxygen-mediated mechanism (EOM) including the electrochemical and electrocatalyst-induced non-electrochemical steps [3,36,37]. The EOM involving HAT includes the electrochemical generation of Ni^3+^-(OH)_ads_ (Ni^2+^ + OH^−^ = Ni^3+^-(OH)_ads_ + e^−^_circuit_) and Ni^3+^-(OH)_ads_–induced HAT (Ni^3+^-(OH)_ads_ + X-H = Ni^2+^ + H_2_O + X·); thereinto, the former is the rate-limiting step in the electrocatalyst function of NOR. During the EOM involving HAT, the proton and electron of the X−H bond in nucleophiles are spontaneously captured by the electrophilic oxygen in Ni^3+^-(OH)_ads_, generated in the electrochemical step, to form X· free radical and H_2_O (Fig. 2c) [35].

### Electrooxidation of primary alcohol on NiO

The NOR system presents a tremendous potential in alcohol electrooxidation [2,13,16–18]. On the NiO electrode, R-CH_2_OH could be electrochemically oxidized to R-COOH (Figs S14 and S15). During the R-CH_2_OH electrooxidation, two processes, i.e. (1) oxidation of R-CH_2_OH to aldehyde (R-CHO) and (2) oxidation of R-CHO to R-COOH, are involved. The electrooxidation of R-CH_2_OH to R-CHO can be explained by the EOM involving HAT. During two Ni^3+^-(OH)_ads_–induced HAT steps, both the hydroxyl group and alpha-methylene (−CH_2_−) in R-CH_2_OH lose a hydrogen atom each to produce R-CHO (Fig. S16). Outwardly, only one oxygen atom is transferred for the electrooxidation of R-CHO to R-COOH, but two Ni^3+^-(OH)_ads_–induced HAT steps should occur in this two-electron transfer process. Hence, there must be a reaction process involving the transfer of two hydrogen atoms and one oxygen atom (maybe H_2_O is involved) during the electrooxidation of R-CHO to R-COOH.

While R-CHO dissolves in water, H_2_O can attack the electrophilic carbonyl carbon in R-CHO to form aldehyde hydrate (geminal diol molecule; R-CH(OH)_2_), which is a hydration reaction [38,39]. By dissolving R-CHO in ^18^O labeled water (H_2_^18^O), almost all oxygen-16 atoms (^16^O) in the aldehyde group were replaced with oxygen-18 atoms (^18^O) from H_2_^18^O within ∼5 minutes (Fig. S17). Hence, the hydration of R-CHO is a spontaneous and reversible reaction, i.e. R-CHO + H_2_O ⇌ R-C(OH)_2_, and it is an electrocatalyst-irrelevant non-electrochemical step in NOR [39]. The oxidation of R-CH_2_OH to R-COOH includes three processes: (1) dehydrogenation of R-CH_2_OH to R-CHO, (2) hydration of R-CHO to R-C(OH)_2_, and (3) dehydrogenation of R-C(OH)_2_ to R-COOH (Fig. S18).

**Figure 3.**
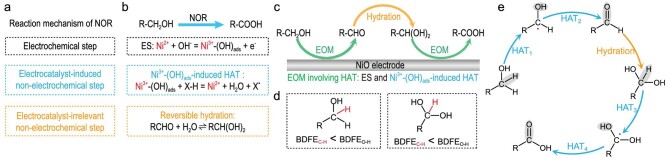
Synergy between the electrochemical and non-electrochemical steps during the electrooxidation of primary alcohol on NiO. (a) Three basic types of elementary steps in NOR. (b) Three basic electrochemical/non-electrochemical steps in the electrooxidation of R-CH_2_OH on NiO. (c) Schematic diagram showing the electrooxidation of R-CH_2_OH on NiO. (d) BDFEs of C–H and O–H bonds in R-CH_2_OH and R-CH(OH)_2_ (R: CH_3_−, CH_3_-CH_2_−, and C_6_H_5_−). (e) Reaction pathway for the electrooxidation of R-CH_2_OH to R-COOH based on non-electrochemical steps.

The NOR mechanism comprises the electrochemical step, electrocatalyst-induced non-electrochemical step, and electrocatalyst-irrelevant non-electrochemical step (Fig. 3a). There are three basic steps for the R-CH_2_OH electrooxidation on NiO (Fig. 3b): (1) electrochemical generation of Ni^3+^-(OH)_ads_, (2) Ni^3+^-(OH)_ads_–induced HAT, and (3) reversible hydration of R-CHO. Thereinto, the EOM involving HAT comprises the electrochemical generation of Ni^3+^-(OH)_ads_ and Ni^3+^-(OH)_ads_–induced HAT. On the NiO electrode, R-CH_2_OH can be electrochemically oxidized to R-COOH due to the synergy between the EOM involving HAT and hydration of R-CHO (Fig. 3c). Although the electrochemical performance of NOR depends on the electrochemical step, the nucleophile oxidation pathway is directly determined by the non-electrochemical steps involving nucleophiles, instead of the electrochemical step.

The free energy of the X–H bond cleavage can be described by the bond dissociation free energy (BDFE) [35,40]. Theoretically, the X–H bond with a lower BDFE is more likely to be broken. According to the DFT calculations of R-CH_2_OH and R-CH(OH)_2_ (R: CH_3_−, CH_3_-CH_2_−, and C_6_H_5_−), the BDFE of the alpha-C-H bond is lower than that of the O–H bond (Figs 3d and S19–S21). Hence, in R-CH_2_OH or R-CH(OH)_2_, the dehydrogenation of the alpha-C–H bond takes precedence over that of the O–H bond. Consequently, we proposed the R-CH_2_OH oxidation pathway based on these five non-electrochemical steps (Figs 3e and S19–S21): (1) dehydrogenation of R-CH_2_OH to R-·CHOH (HAT_1_), (2) dehydrogenation of R-·CHOH to R-CHO (HAT_2_), (3) hydration of R-CHO to R-CH(OH)_2_, (4) dehydrogenation of R-CH(OH)_2_ to R-·C(OH)_2_ (HAT_3_), and (5) dehydrogenation of R-·C(OH)_2_ to R-COOH (HAT_4_).

### Electrophilic oxygen-mediated mechanism involving C–C bond cleavage

**Figure 4.**
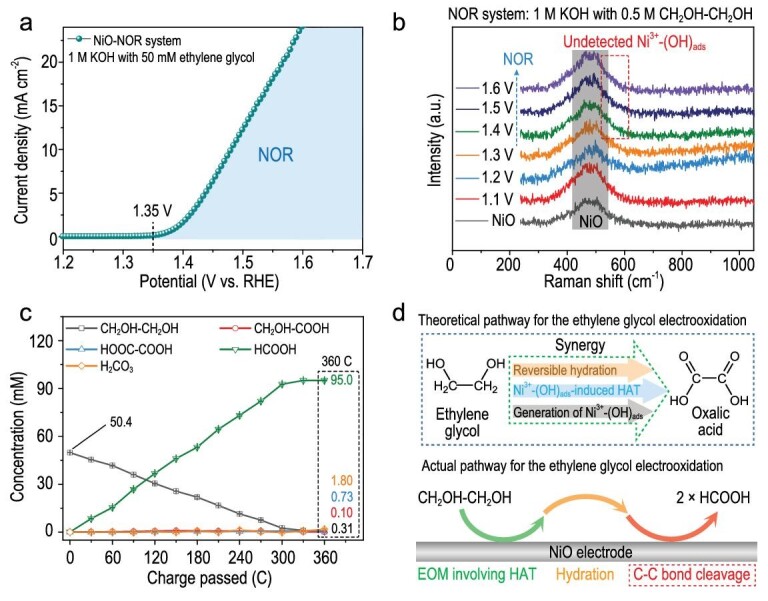
Electrooxidation of CH_2_OH-CH_2_OH on NiO. (a) Anodic polarization curve of NiO in 1 M KOH with 50 mM CH_2_OH-CH_2_OH. (b) *In situ* Raman spectra of NiO electrode in 1 M KOH with 0.5 M CH_2_OH-CH_2_OH. (c) Concentration changes of CH_2_OH-CH_2_OH and its electrooxidation products versus charge passed during the electrooxidation of CH_2_OH-CH_2_OH on NiO. (d) Schematic diagram illustrating the theoretical/actual pathway for the CH_2_OH-CH_2_OH electrooxidation on NiO.

The NiO electrode cannot catalyze the C–C bond cleavage during the R-CH_2_OH electrooxidation due to two main reasons, i.e. (1) the limited electrophilicity (oxidizability) of Ni^3+^-(OH)_ads_, and (2) the inert C–C bond in R-CH_2_OH. For vicinal diols (R-CHOH-CHOH), two hydroxyl groups (electron-withdrawing group) significantly reduce the bond dissociation energy of the C–C bond so that the C–C bond cleavage can be catalyzed during the R-CHOH-CHOH electrooxidation on NiO [10,16–18]. On the NiO electrode, the ethylene glycol (CH_2_OH-CH_2_OH; the simplest vicinal diol) electrooxidation occurs at a potential above ∼1.35 V (Fig. 4a). During the CH_2_OH-CH_2_OH electrooxidation on NiO, Ni^3+^-(OH)_ads_ can be generated, but cannot be accumulated due to the EOM, thereby yielding the undetectable Ni^3+^-(OH)_ads_ (Fig. 4b). According to analysis of these products, one CH_2_OH-CH_2_OH molecule can be electrochemically oxidized to two HCOOH molecules on the NiO electrode, accompanied by the C–C bond cleavage (Figs 4c and S22). If the reaction mechanism of the CH_2_OH-CH_2_OH electrooxidation is the same as that of the R-CH_2_OH electrooxidation, the electrooxidation product of CH_2_OH-CH_2_OH should be oxalic acid (COOH-COOH), instead of HCOOH (Fig. 4d). There must be a special process involving the C–C bond cleavage for the CH_2_OH-CH_2_OH electrooxidation on NiO (Fig. S23).

Among three C–C bond cleavage types, the oxidative C–C bond cleavage is the only possible means during alcohol electrooxidation since non-spontaneous reduction reactions cannot occur in the NOR system (Fig. S24) [41–43]. For the NOR on NiO, it is challenging to distinguish the oxidative C–C bond cleavage process from other electrochemical/non-electrochemical steps [44]. To avoid the irrelevant non-electrochemical steps (i.e. Ni^3+^-(OH)_ads_–induced HAT and hydration of R-CHO), the alpha ketonic acid (R-CO-COOH) without hydroxyl/aldehyde group was used in the NOR system for studying the oxidative C–C bond cleavage (Fig. S25) [45].

**Figure 5.**
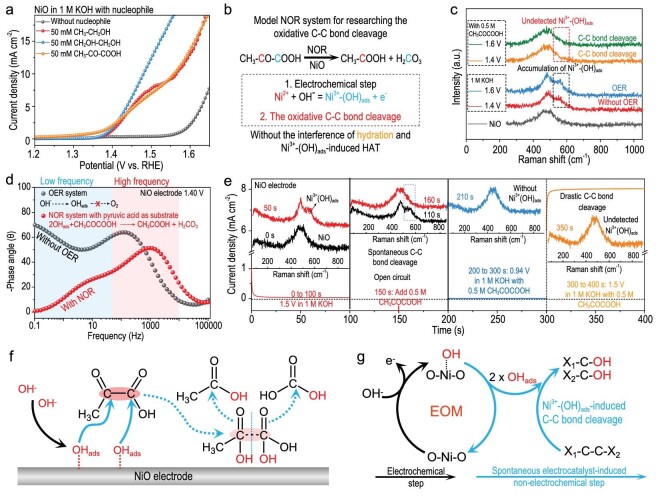
Electrochemical oxidation-induced C–C bond cleavage on NiO. (a) Anodic polarization curves of NiO in 1 M KOH with/without nucleophiles (CH_3_-CH_2_OH, CH_2_OH-CH_2_OH, and CH_3_-CO-COOH). (b) Model NOR system for researching oxidative C–C bond cleavage on NiO: electrooxidation of CH_3_-CO-COOH. (c and d) *In situ* Raman spectra (c) and Bode plots (d) of NiO in 1 M KOH with/without 0.5 M CH_3_-CO-COOH. (e) Current density versus time for the NiO electrode (0 to 100 s: 1.5 V; 100 to 200 s: open-circuit condition; 200 to 300 s: 0.94 V; 300 to 400 s: 1.5 V) in different electrolytes (0 to 150 s: 1 M KOH; 150 to 400 s: 1 M KOH with 0.5 M CH_3_-CO-COOH), inset: Synchronous *in situ* Raman spectra. (f and g) Schematic diagrams illustrating the CH_3_-CO-COOH electrooxidation on NiO (f) and the EOM involving C–C bond cleavage (g).

The electrooxidation of pyruvic acid (CH_3_-CO-COOH; the model compound for R-CO-COOH) on NiO occurs at a potential above ∼1.35 V (which is similar to other NOR systems), and CH_3_-CO-COOH can be electrochemically oxidized to CH_3_-COOH and carbonic acid (H_2_CO_3_) (Figs 5a and S26). Hence, the CH_3_-CO-COOH electrooxidation on NiO involves two basic steps: (1) the electrochemical generation of Ni^3+^-(OH)_ads_ and (2) oxidative C–C bond cleavage of CH_3_-CO-COOH (Fig. 5b). On the NiO electrode, the generated Ni^3+^-(OH)_ads_ can react with CH_3_-CO-COOH to generate CH_3_-COOH and H_2_CO_3_ so that the Ni^3+^-(OH)_ads_ cannot be detected by *in situ* Raman spectroscopy (Figs 5c and S27). For example, the accumulated Ni^3+^-(OH)_ads_ cannot be oxidized to oxygen on the NiO electrode at 1.40 V in the OER system; however, for the NOR system containing CH_3_-CO-COOH at 1.40 V, the Ni^3+^-(OH)_ads_ can catalyze oxidative C–C bond cleavage at the low-frequency interface (Figs 5c, d, S28 and S29).

To further understand the reaction mechanism between the C–C bond and Ni^3+^-(OH)_ads_, the electrochemical generation of Ni^3+^-(OH)_ads_ and the oxidative C–C bond cleavage of CH_3_-CO-COOH should be separated (Figs 5e and S30). Ni^3+^-(OH)_ads_ was formed and accumulated at 1.50 V in 1 M KOH (0 to 100 s), and the generated Ni^3+^-(OH)_ads_ still existed in 1 M KOH under an open-circuit condition (100 to 150 s); however, after adding 0.5 M CH_3_-CO-COOH into 1 M KOH at 150 s, the Ni^3+^-(OH)_ads_ was consumed completely under the open-circuit condition (150 to 200 s), accompanied by the oxidative C–C bond cleavage (Fig. 5e). Hence, CH_3_-CO-COOH can spontaneously react with Ni^3+^-(OH)_ads_ to generate CH_3_-COOH and H_2_CO_3_, and this process is defined as the Ni^3+^-(OH)_ads_–induced C–C bond cleavage, which is an electrocatalyst-induced non-electrochemical step.

We consider that two Ni^3+^-(OH)_ads_ intermediates attack the C–C bond in CH_3_-CO-COOH to form two C–OH bonds, thereby yielding the spontaneous C–C bond cleavage (Fig. 5f) [46]. Hence, in addition to the EOM involving HAT, there is another electrocatalyst function for the NOR on NiO, i.e. the EOM involving C–C bond cleavage including the electrochemical generation of Ni^3+^-(OH)_ads_ and Ni^3+^-(OH)_ads_–induced C–C bond cleavage (Figs 5g and S31). The EOM involving C–C bond cleavage is accompanied by the formation of the O-H bond, and the C–C bond cleavage products of a hydroxymethyl group (–CH_2_OH), aldehyde group (–CHO), carboxyl group (–COOH), secondary hydroxyl group (R-CHOH–), and ketone group (R-CO–) are formaldehyde hydrate (H_2_C(OH)_2_), HCOOH, H_2_CO_3_, aldehyde hydrate (R-CH(OH)_2_), and R-COOH, respectively (Fig. S32). Generally, there are two electrocatalyst functions, i.e. (1) the EOM involving HAT and (2) EOM involving C–C bond cleavage. According to the density functional theory (DFT) calculations, the electrochemical generation of Ni^3+^-(OH)_ads_ is the rate-limiting step in these two EOMs, because Ni^3+^-(OH)_ads_ can spontaneously catalyze HAT or C–C bond cleavage (Fig. S33).

### Electrooxidation of vicinal diol on NiO

**Figure 6.**
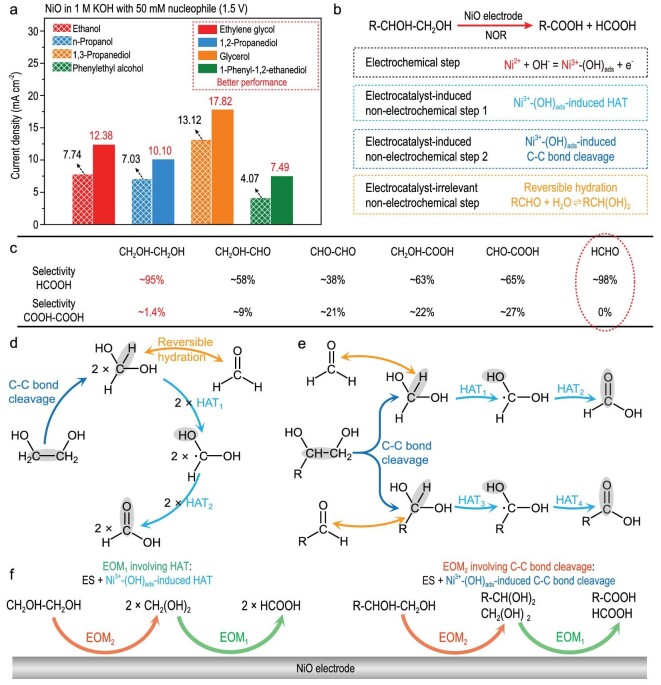
Synergy between the electrochemical and non-electrochemical steps during the electrooxidation of vicinal diol on NiO. (a) Current densities (at 1.5 V) for the electrooxidations of different primary alcohols and vicinal diols on NiO. (b) Basic steps for the electrooxidation of R-CHOH-CH_2_OH to R-COOH and HCOOH on NiO. (c) Selectivities of HCOOH and COOH-COOH for the electrooxidations of HCHO, CHO-COOH, CH_2_OH-COOH, CHO-CHO, CH_2_OH-CHO, and CH_2_OH-CH_2_OH on NiO. (d and e) Reaction pathways of the electrooxidations of CH_2_OH-CH_2_OH (d) and R-CHOH-CH_2_OH (e) based on non-electrochemical steps. (f) Schematic diagram illustrating the electrooxidation of CH_2_OH-CH_2_OH or R-CHOH-CH_2_OH on NiO.

Compared with the R-CH_2_OH electrooxidation, the NiO electrode exhibits better electrochemical performances for the R-CHOH-CH_2_OH electrooxidation because of more reaction sites in R-CHOH-CH_2_OH (Figs 6a and S34). According to analysis of the products, R-CHOH-CH_2_OH can be electrochemically oxidized to R-COOH and HCOOH accompanied by the C–C bond cleavage (Fig. S35). As for polyhydric alcohols, multiple C–C bonds in polyhydric alcohols can be broken during NOR; e.g. one glycerol (CH_2_OH-CHOH-CH_2_OH) molecule can be electrochemically oxidized to three HCOOH molecules during the electrooxidation over NiO [16–18]. We further prove that the R-CHOH-CH_2_OH oxidation accompanied by the C–C bond cleavage (e.g. the oxidation of CH_2_OH-CH_2_OH to HCOOH) can be spontaneously catalyzed by Ni^3+^-(OH)_ads_ without an applied voltage (Fig. S36). There may be four basic steps in the electrooxidation of R-CHOH-CH_2_OH to R-COOH and HCOOH on NiO, i.e. (1) the electrochemical generation of Ni^3+^-(OH)_ads_, (2) Ni^3+^-(OH)_ads_–induced HAT, (3) Ni^3+^-(OH)_ads_–induced C–C bond cleavage, and (4) hydration of R-CHO (Fig. 6b). Considering the CH_2_OH-CH_2_OH (the simplest vicinal diol) electrooxidation as an example, the possible electrooxidation products are COOH-COOH, HCOOH, and H_2_CO_3_. The sluggish deep electrooxidation of HCOOH to H_2_CO_3_ on NiO can influence the concentrations of HCOOH and H_2_CO_3_ during the CH_2_OH-CH_2_OH electrooxidation (Fig. S37). Hence, the COOH-COOH selectivity is an important piece of evidence to identify the CH_2_OH-CH_2_OH electrooxidation pathway.

According to analyses performed on the electrooxidations of all possible reaction intermediates (i.e. HCHO, CHO-COOH, CH_2_OH-COOH, CHO-CHO, and CH_2_OH-CHO) on NiO, we estimated the probabilities of the basic reaction processes for the CH_2_OH-CH_2_OH electrooxidation on NiO through a backward induction strategy (Figs S38–S44) [47]. Thereinto, only the HCHO electrooxidation exhibits a higher HCOOH selectivity (∼98%) than that attained from the CH_2_OH-CH_2_OH electrooxidation (∼95%), and the COOH-COOH selectivities for the electrooxidations of other possible reaction intermediates (i.e. CHO-COOH, CH_2_OH-COOH, CHO-CHO, and CH_2_OH-CHO) far outweigh that for the CH_2_OH-CH_2_OH electrooxidation (∼1.4%) (Fig. 6c). These results demonstrate that the EOM involving C–C bond cleavage takes priority during the CH_2_OH-CH_2_OH electrooxidation on NiO, and the dominant reaction intermediate is CH_2_(OH)_2_, rather than other C_2_ intermediates (Fig. S44).

According to the BDFEs of CH_2_(OH)_2_ or R-CH(OH)_2_, the dehydrogenation of the alpha–C–H bond takes precedence over that of the O-H bond (Fig. S45). The CH_2_OH-CH_2_OH electrooxidation pathway on NiO is described as follows (Fig. 6d): (1) CH_2_OH-CH_2_OH is oxidized to two CH_2_(OH)_2_ molecules during the Ni^3+^-(OH)_ads_–induced C–C bond cleavage; (2) The C–H and O–H bonds in CH_2_(OH)_2_ undergo dehydrogenation in turn to form HCOOH during two Ni^3+^-(OH)_ads_–induced HAT steps. Both the EOM involving C–C bond cleavage and EOM involving HAT work during the R-CHOH-CH_2_OH electrooxidation on NiO (Fig. 6e and f). In the first stage, R-CHOH-CH_2_OH is electrochemically oxidized to R-CH_2_(OH)_2_ and CH_2_(OH)_2_*via* the EOM involving C–C bond cleavage. In the second stage, due to the EOM involving HAT, the alpha-C–H and O–H bonds in CH_2_(OH)_2_ or R-CH_2_(OH)_2_ undergo dehydrogenation in turn to form HCOOH or RCOOH, respectively.

## CONCLUSION

In this study, we investigated the alcohol (i.e. R-CH_2_OH and R-CHOH-CH_2_OH) electrooxidation on NiO. The alcohol electrooxidation on NiO is an indirect electrooxidation reaction with the Ni^3+^-(OH)_ads_ containing electrophilic oxygen as the redox mediator. The electrochemical step is the electrochemical generation of Ni^3+^-(OH)_ads_. Ni^3+^-(OH)_ads_–induced HAT and Ni^3+^-(OH)_ads_–induced C–C bond cleavage are electrocatalyst-induced non-electrochemical steps. Hence, there are two EOMs including the electrochemical step and electrocatalyst-induced non-electrochemical step, i.e. (1) the EOM involving HAT and (2) EOM involving C–C bond cleavage. The hydration of R-CHO is an electrocatalyst-irrelevant non-electrochemical step. The NOR system depends on the synergy between the electrochemical and non-electrochemical steps. The synergy between EOM involving HAT and hydration of R-CHO results in the electrooxidation of R-CH_2_OH to R-COOH on NiO. The synergy between EOM involving HAT and EOM involving C–C bond cleavage causes the electrooxidation of R-CHOH-CH_2_OH to R-COOH and HCOOH on NiO. As such, this study establishes a unified NOR mechanism for alcohol electrooxidation, which will open up new possibilities for developing NOR. Our work highlights the synergy between the electrochemical and non-electrochemical steps in the NOR system. We firmly believe that, by tuning electrochemical and non-electrochemical steps, the NOR system can achieve various aqueous organic electrooxidation reactions toward the green synthesis of high-value organic compounds.

## MATERIALS AND METHODS

Ni(NO_3_)_2_·6H_2_O, NaOH, and KOH were purchased from Sinopharm Chemical Reagent Co., Ltd. 5 wt% Nafion solution was purchased from DuPont™. All organic compounds were purchased from Sigma-Aldrich.

The morphologies and crystalline structures of electrocatalysts were identified by scanning electron microscope (SEM; Hitachi S-4800, Hitachi Corporation, Japan), transmission electron microscope (TEM; FEI Tecnai G20, FEI Company, USA), X-ray diffraction (XRD; Bruker D8 Advance diffractometer, Bruker, Germany). The Ni K-edge X-ray absorption was measured using Taiwan Photon Source (TPS) Quick-scanning X-ray absorption spectroscopy beamline 44A1 under ambient pressure at the National Synchrotron Radiation Research Center (NSRRC), Hsinchu. XAS measurement was made in transmission mode using ion chamber detectors. And pure cobalt metal foil was used for energy calibration. All spectra were carried out with 1 Hz oscillating frequency for 2 min. We have 240 spectra to average and increase the S/N ratio and normalize unit step height in the absorption coefficient from well below to well above the edges. All X-ray absorption spectroscopy (XAS) spectra were aligned, merged, deglitched, and normalized using the Athena (version number 0.9.26) module implemented in the IFEFFIT software packages. The electrode surface species was identified by X-ray photoelectron spectroscopy (XPS; Axis Supra, Kratos Company, England) and Raman spectrum (Alpha300R, WETEC, Germany). The XPS spectra were calibrated by adventitious carbon (C1s at 284.8 eV).

### Preparation of NiO

The NiO hexagonal nanosheets were synthesized with the β-Ni(OH)_2_ hexagonal nanosheets as precursor. Then 5 mmol Ni(NO_3_)_2_·6H_2_O was dissolved in 30 mL deionized water. The Ni(NO_3_)_2_ solution was mixed with 2 M NaOH solution, thus obtaining the β-Ni(OH)_2_ suspension (pH: ∼13.5). The β-Ni(OH)_2_ suspension was transferred into a 100-mL Teflon-lined stainless-steel autoclave, and the autoclave was placed in an oven at 160°C for 6 h. After cooling, the synthesized β-Ni(OH)_2_ nanosheets were repeatedly washed by deionized water and anhydrous ethanol, then dried in a vacuum drying chamber for 10 h at 60°C. Finally, the β-Ni(OH)_2_ hexagonal nanosheets were calcined under an air atmosphere at 400°C for 2 h (the heating/cooling rate: 5°C min^−1^), thus synthesizing the NiO hexagonal nanosheets.

## Supplementary Material

nwad099_Supplemental_File
