# Supplementary material for: Unraveling the electrophilic oxygen-mediated mechanism for alcohol electrooxidation on NiO
Source: Natl Sci Rev. 2023 Apr 13;10(5):nwad099. doi: 10.1093/nsr/nwad099 (PMC10243987; doi:10.1093/nsr/nwad099)
Supplement: nwad099_Supplemental_File [file nwad099_supplemental_file.pdf]

Supplementary data

Unraveling the electrophilic oxygen-mediated mechanism for alcohol electrooxidation on NiO

Wei Chen[1,†], Jianqiao Shi[1,†], Chao Xie[1], Wang Zhou[2], Leitao Xu[1], Yingying Li[1], Yandong Wu[1], Binbin Wu[1], Yu-Cheng Huang[3], Bo Zhou[1], Ming Yang[1], Jilei Liu[2], Chung-Li Dong[3], Tehua Wang[1], Yuqin Zou[1,4,*], and Shuangyin Wang[1,4,*]

[1]State Key Laboratory of Chemo/Bio-Sensing and Chemometrics, College of Chemistry and Chemical Engineering, Advanced Catalytic Engineering Research Center of the Ministry of Education, Hunan University, Changsha, Hunan, 410082
[2]College of Materials Science and Engineering, Hunan University, Changsha, Hunan, 410082
[3]Research Center for X-ray Science & Department of Physics, Tamkang University, 151 Yingzhuan Rd., New Taipei City 25137
[4]Shenzhen Institute of Hunan University, Shenzhen, Guangdong, 518057

***Corresponding authors.** E-mails: yuqin_zou@hnu.edu.cn; shuangyinwang@hnu.edu.cn

†Equally contributed to this work.

Methods

Electrochemical measurements. All electrochemical measures were carried out in the three-electrode system with the thermostatic water bath (25 ℃). The carbon rod and Hg/HgO electrode were adopted as counter and reference electrodes, respectively. All electrochemical data was converted to the reversible hydrogen electrode (RHE). The reversible hydrogen electrode (RHE) was determined with platinum gauze electrode as work electrode in the hydrogen-saturated electrolyte solution. All anodic polarization curves were iR-corrected according to the following equation: $E_{iR\text{-}corrected} = E - I \times Rs$. Electrochemical impedance spectroscopy (EIS) was performed by Autolab PGSTAT302N (Eco Chemie, Utrecht, Netherlands) with the frequency range from 1 M Hz to 0.1 Hz. Other electrochemical measurements were carried out by the electrochemical workstation (CHI 760D, CH Instruments Ins, Shanghai). All electrodes (0.2 $mg_{catalyst}$ cm^{-2}) were prepared by drop-casting method. The electrocatalyst ink is composed of 4 mg electrocatalyst, 450 μL deionized water, 500 μL ethanol, and 50 μL Nafion solution (5 wt%). Most electrochemical measurements were carried out by using glassy carbon electrode (0.196 cm^2), such as anodic polarization curves and EIS. For the identification and quantification of reaction intermediates and products in NOR system, NiO was painted on carbon paper (CP; 1 cm^2) in order to increase the electrolysis efficiency. For the characterization of electrode (such as Raman and XPS spectra), the glassy carbon plate electrode (1 cm^2) was adopted, and the electrocatalyst ink without Nafion (including 4 mg electrocatalyst, 500 μL deionized water, and 500 μL ethanol) was carried out in order to avoid the influence of Nafion on these characterizations. The electrooxidation of nucleophile over NiO/CP electrode for the product identification and quantification were conducted in 10 mL of 1 M KOH solution with 50 mM nucleophile.

Identification and quantification of electrooxidation products. Nucleophiles and their electrooxidation products were identified by 1H nuclear magnetic resonance (1H NMR; Bruker Avance III 400 MHz, Bruker Co., Germany). Nucleophiles and their electrooxidation products were quantified by gas chromatograph-mass spectrometer instrument (GC-MS; GC2010-MS, Shimadzu, Japan) and high performance liquid chromatography instrument (HPLC; LC 20A, Shimadzu, Japan). Thereinto, R-CH_2OH (such as ethanol, n-butyl alcohol, and benzyl alcohol) and R-COOH (such as acetic acid, propionic acid, and benzoic acid) were quantified by the GC-MS. Other small organic molecules were quantified by the HPLC. We used the HPLC instrument equipped with a Coregel-87H3 column (7.8 mm × 300 mm), and the refractive index detector was carried out to identify small organic molecules (alcohols, aldehydes, and carboxylic acids). The mobile phase is 5×10^{-3} M H_2SO_4 aqueous solution with a constant flow rate of 0.5 mL min^{-1}, and the column temperature is 60 ℃. In this work, the yield of H_2CO_3 was indirectly quantified based on the conservation of the total amount of carbon in the NOR system.

The conversion rate, selectivity, and Faradic effificiency for NOR are as follows:

Conversion rate (%) = (mol of nucleophile after electrolysis) / (mol of initial nucleophile) × 100%

Selectivity (%) = (mol of product) / (mol of consumed nucleophile) × 100%

Faradic effificiency (%) = (mol of product) × n × F / (total passed charge) × 100%

n = 2 for the electrooxidation of R-CH_2OH to R-CHO, the electrooxidation of R-CHO/R-$CH(OH)_2$ to R-COOH, and the oxidative C-C bond cleavage (such as the electrooxidation of R-CO-COOH to R-COOH and HCOOH).

n = 4 for the electrooxidation of R-CH_2OH to R-COOH.
n = 6 for the electrooxidation of R-CHOH-CH_2OH to R-COOH and HCOOH.
n = 8 for the electrooxidation of CH_2OH-CHOH-CH_2OH to HCOOH.
F is the Faraday constant (96485 C mol^{-1}).

Theoretical methods. NOR is a complex reaction including the electrochemical step and spontaneous non-electrochemical process. Therefore, the electrochemical step and spontaneous non-electrochemical process should be separately calculated by density functional theory (DFT).
For the electrochemical generation of Ni^{3+}-$(OH)_{ads}$ on NiO, the calculations of the spin-polarized DFT were implemented by using the Vienna Ab-initio Simulation Package (VASP) [1, 2]. The Perdew-Burke-Ernzerhof (PBE) functional and projector-augmented-wave (PAW) pseudopotentials were used for exchange-correlation energy functional and core-valence interactions, respectively [3]. DFT + U method was used for Ni^{2+} ions (U = 6.45eV for NiO) [4]. The cutoff energy of 600 eV was carried out for the optimization of the stress tensor and atomic forces of NiO (space group: Fm-3m) bulk. The cutoff energy of 400 eV was carried out for calculating NiO (200) surface. The Gaussian smearing method with a width of 0.05 eV was applied. The NiO (200) surface was built as (2×2) supercell with four atomic layers. The bottom two atomic layers were fixed, and topmost two atomic layers and adsorbed species were relaxed. Brillouin zone was sampled with a Gamma-centered k-point grid. The periodic models were constructed with a vacuum space of 15 Å. The D3 van der Waals correction was employed in the calculations. The energy tolerance and maximum force tolerance were set to 1×10^{-5} eV and 0.05 eV/Å, respectively. Implicit solvation was added using VASPsol. The traditional computational hydrogen electrode was used to determine the reaction energies. To calculate the adsorption of OH^- on the catalyst surface ($OH^- + * \rightarrow OH^* + e^-$), we used $H^+ + OH^- \leftrightarrow H_2O$ to avoided the direct calculation of OH^-. Note that the computation assessment of charged species (such as OH^-) is currently difficult. The free energy of OH^- adsorption on NiO (200) surface is calculated as $\Delta G(OH^*) = G(OH^*) - G(H_2O,l) - G(*) + 0.5G(H_{2},g) - eU - 0.0592pH$. Here, U=0 and pH=0 in the system.

On the other hand, for the non-electrochemical steps (such as HAT and hydration), all calculations were carried out using Gaussian 16 program. The structures of organic molecules were optimized at M06-2X/def2-tzvp level with solvent effects accounted by the SMD. Harmonic vibrational frequencies were calculated to identify all stationary points to be either minima (with zero imaginary frequencies) or transition states (with one imaginary frequency). Selected transition states have been checked by intrinsic reaction coordinate (IRC) calculations.

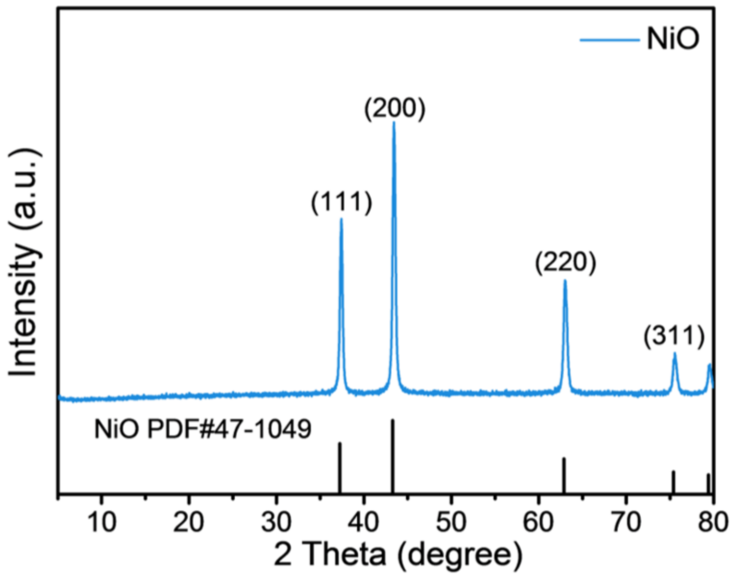

Figure S1. X-ray diffraction (XRD) pattern of NiO. All peaks of the NiO sample accord with the NiO phase (PDF#47-1049).

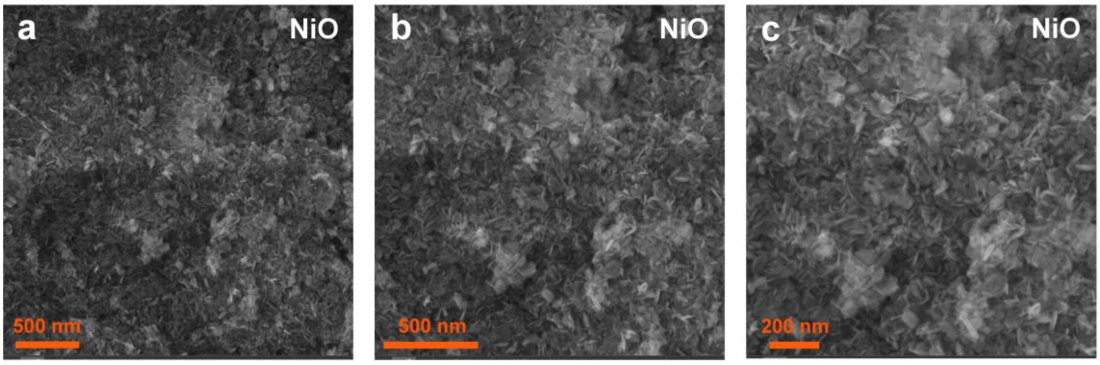

Figure S2. Scanning electron microscopy (SEM) images of NiO nanosheets.

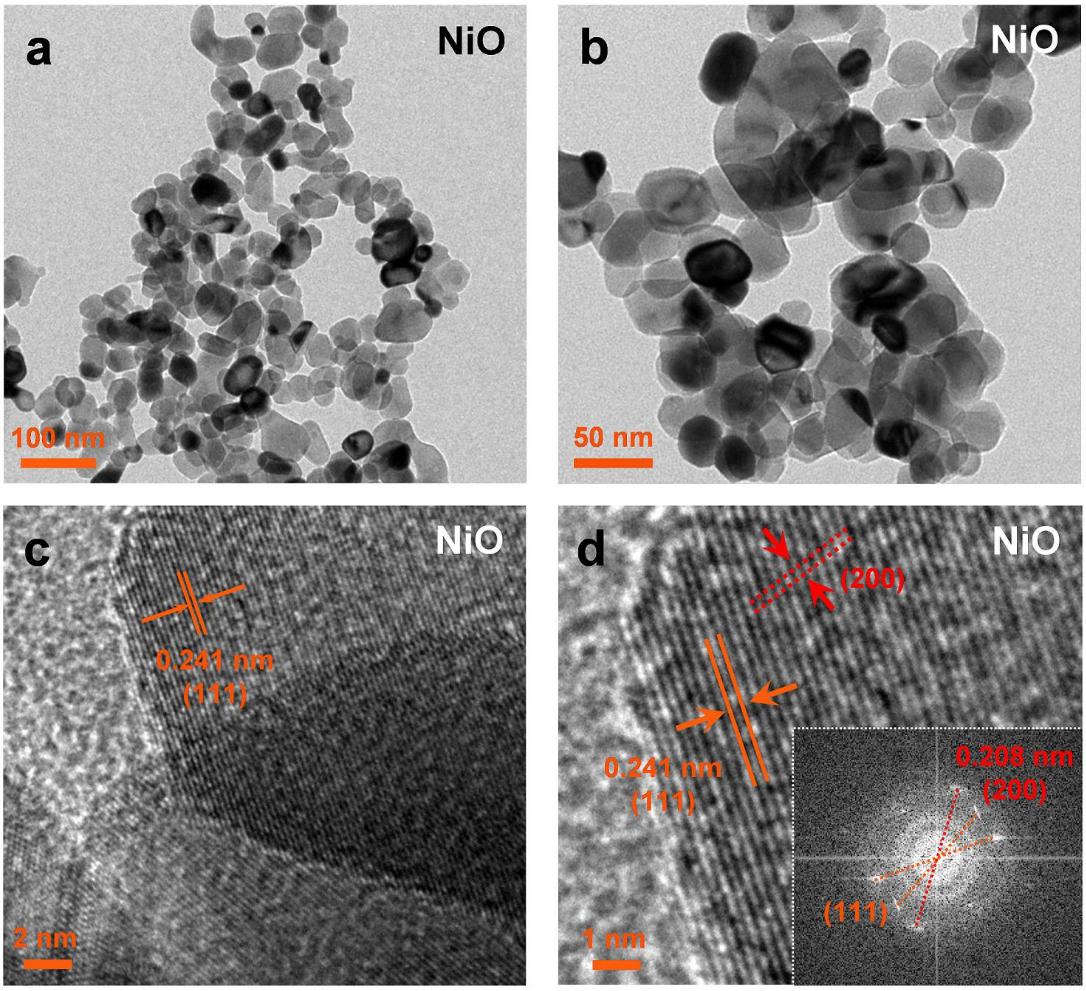

Figure S3. Transmission electron microscopy (TEM) images of NiO nanosheets.

The NiO sample is composed of hexagonal nanosheets with the lateral size of 40-60 nm. As shown in Fig. 1c, the lattice spacing of 0.241 nm, corresponding to the distance of (111) lattice plane of NiO, can be observed in the TEM image. Because of the fuzzy imaging of atoms, the TEM image shows a blurry (200) lattice plane of NiO. According to the selected area electron diffraction (SAED) pattern, we can observe the (200) lattice plane (the lattice spacing of 0.208 nm) on the NiO nanosheet.

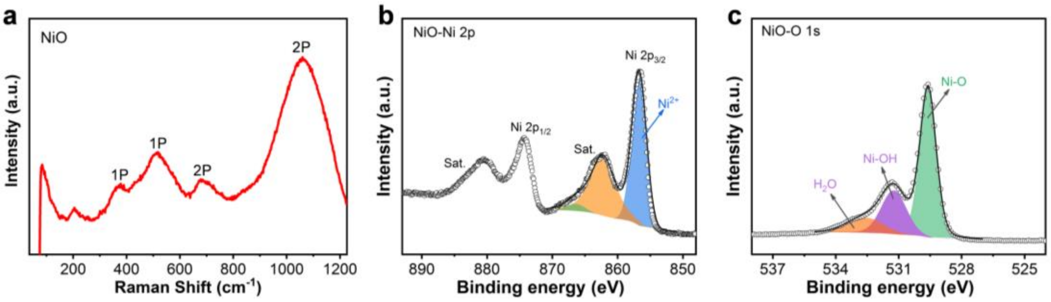

Figure S4. (a) Raman of the NiO sample. (b) Curve-resolved XPS of the Ni 2p region for the NiO sample. (c) Curve-resolved XPS of the O 1s region for the NiO sample.

For the Raman spectrum of NiO, the Raman peaks at 390 and 530 cm^{-1} are corresponding to one-phonon (1P) of NiO vibrational modes, and the Raman peaks at 690 and 1090 cm^{-1} are corresponding to two-phonon (2P) transverse optical (TO) and 2P longitudinal optical (LO) of NiO vibrational modes, respectively. The XPS spectra of NiO at Ni $2p_{2/3}$ are fitted by employing three components: Ni^{2+} species (peak at 856.70 eV) and two satellite peaks. The XPS spectra of NiO at O 1s are fitted by employing three components: adsorbed H_2O (peak at 532.60 eV), Ni-OH (531.20 eV), and Ni-O (peaks at 529.60 eV), respectively. These results prove that the NiO sample was synthesized successfully.

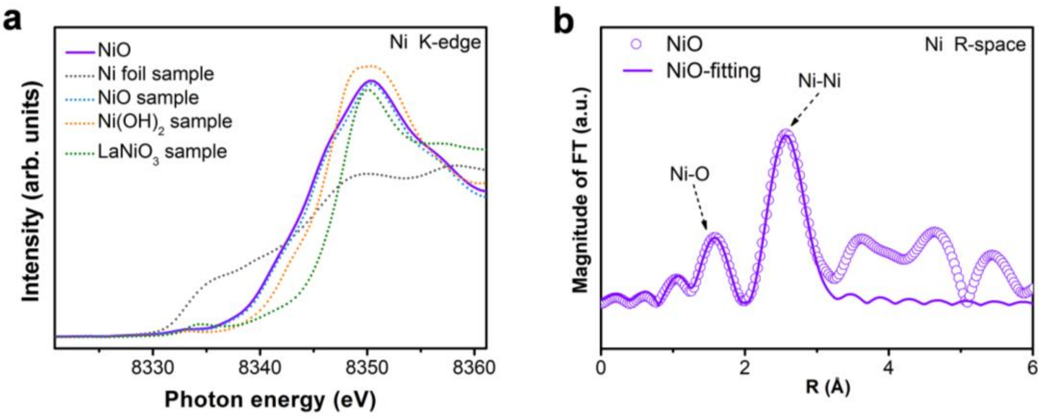

c

Sample	Bond	N	$Sigma^2$	E_0 (eV)	reff	Delta_R	R (Å)
Ni-O	Ni-O	5.052	0.00842	-5.488	2.00	0.08327	2.083
	Ni-Ni	11.532	0.00841	-5.564	2.90	0.05969	2.959

Figure S5. (a) Ni K-edge X-ray absorption near edge structure (XANES) spectrum for NiO. (b) Fourier transform of the Ni/Fe K-edge EXAFS oscillations and EXAFS curve-fitting results for NiO. (c) Table of EXAFS fitting results for NiO.

According to X-ray absorption spectroscopy (XAS) characterization, the crystal structure of NiO was further confirmed.

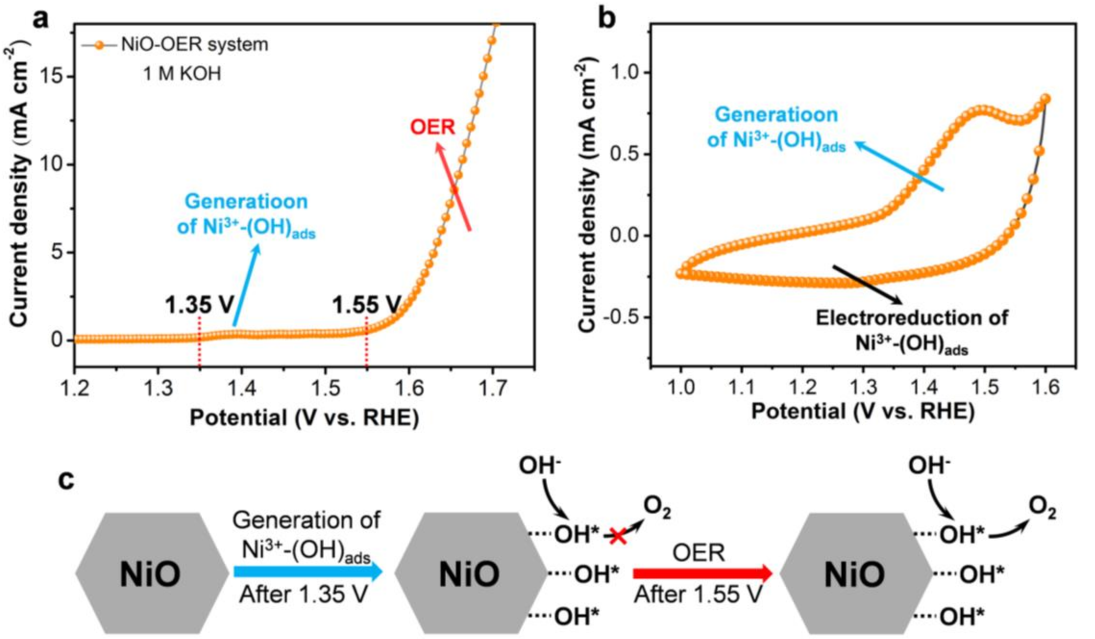

Figure S6. (a) Anodic polarization curve of NiO in the NOR system (1 M KOH). (b) Cyclic voltammetry (CV) curve of the NiO electrode in 1 M KOH with the scan rate of 50 mV s^{-1}. (c) Schematic illustration of the surface species evolution for the NiO electrode in the OER system.

The CV curve shows the redox reaction of the NiO electrode in 1 M KOH, including the electrochemical generation of Ni^{3+}-$(OH)_{ads}$ and the electroreduction of Ni^{3+}-$(OH)_{ads}$. There is no electrochemical reaction for the NiO electrode in the OER system at a potential below ~1.35 V. For the NiO electrode in the OER system, Ni^{3+}-$(OH)_{ads}$ can be generated at a potential above ~1.35 V, and the generated Ni^{3+}-$(OH)_{ads}$ cannot be further oxidized to oxygen at a potential below ~1.55 V.

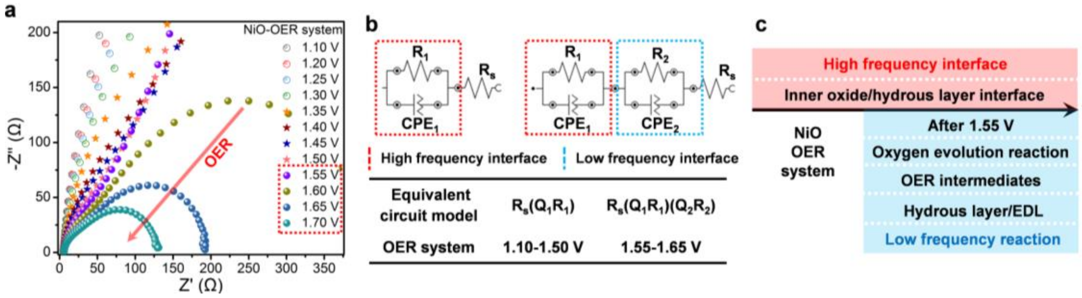

Figure S7. Nyquist (a), equivalent circuit models (b), and potential-dependent behavior (c) for the NiO electrode in OER system.

The corresponding EIS-fitting result is listed in Supplementary Table 1. There is only one semicircle at Nyquist plots of the NiO electrode in OER system at 1.10 to 1.50 V; therefore, the $R_s(Q_1R_1)$ model was employed. There are two semicircles at Nyquist plots of the NiO electrode in OER system at a potential above 1.55 V; hence, the $R_s(Q_1R_1)(Q_2R_2)$ model was employed. The parallel Q_1R_1 and Q_2R_2 elements are closely linked to the high and low frequency interfaces, respectively. For the NiO electrode, the high frequency interface is between inner oxide layer and surface hydrous layer, and the low frequency interface is between surface hydrous layer and electrical double layer (EDL) [5, 6].

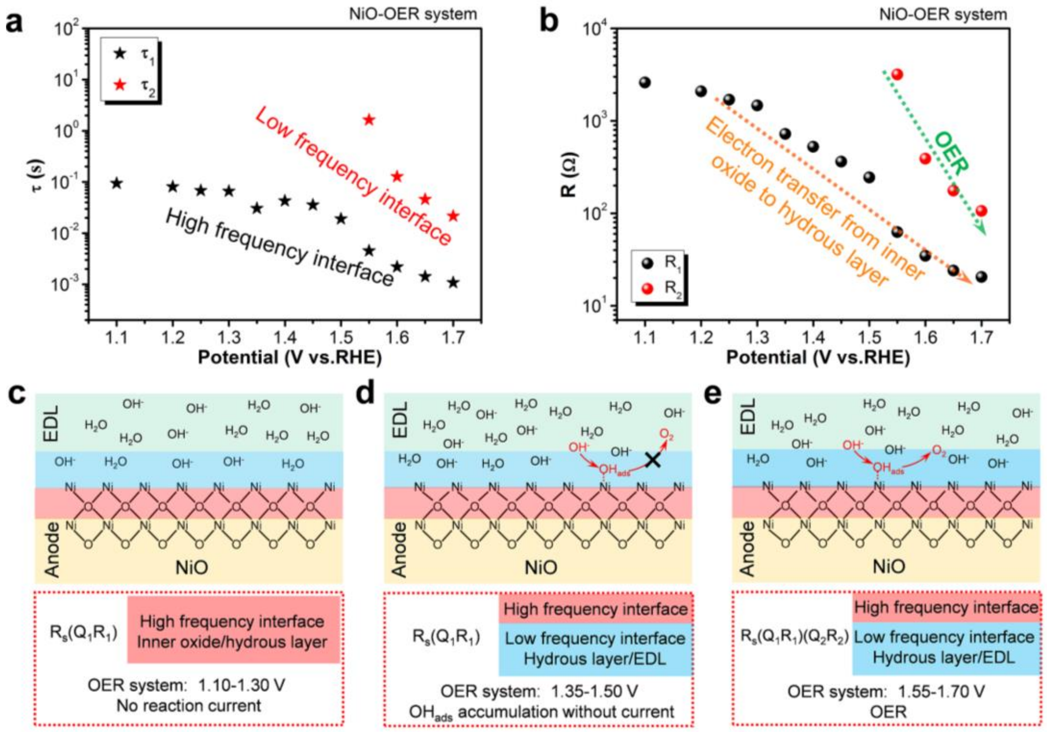

Figure S8. (a) Time constants (τ_1 and τ_2) versus potential for NiO electrode in OER system. (b) Equivalent resistances (R_1 and R_2) versus potential for NiO electrode in OER system. (c-e) Schematic diagrams showing the OER system of NiO at different potential ranges.

As the potential increases for the NiO electrode in OER system, the ever-decreasing equivalent resistance of R_1 is corresponding to the increasing electrical conductivity of the high frequency interface. The second semicircle (R_2) appears at a potential above 1.55 V, and the equivalent resistance of R_2 decreases as the potential increases, corresponding to OER. At 1.35 to 1.50 V, the hydroxyl ion can be electrochemically adsorbed on Ni^{3+} site, but cannot be oxidized to oxygen, leading to the accumulation of Ni^{3+}-$(OH)_{ads}$ on NiO electrode. At a potential above 1.55 V, the OH_{ads} in Ni^{3+}-$(OH)_{ads}$ can be electrochemically oxidized to oxygen (OER) at the low frequency interface between surface hydrous layer and EDL[3].

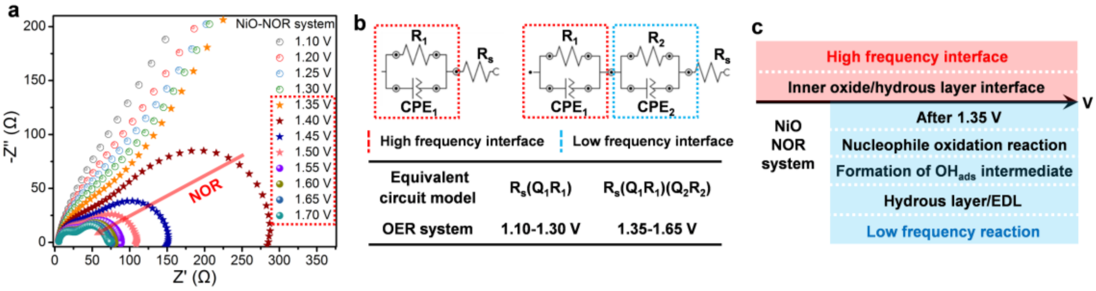

Figure S9. Nyquist (a), equivalent circuit models (b), and potential-dependent behavior (c) for the NiO electrode in the NOR system (1 M KOH with 0.5 M ethanol).

The corresponding EIS-fitting result is listed in Supplementary Table 2. There is only one semicircle at Nyquist plots at 1.10 to 1.30 V. There are two semicircles at a potential above 1.35 V. Therefore, the $R_s(Q_1R_1)$ and $R_s(Q_1R_1)(Q_2R_2)$ models were employed for the NOR system of NiO electrode at the potential range from 1.10 to 1.30 V and the potential range from 1.35 to 1.70 V, respectively.

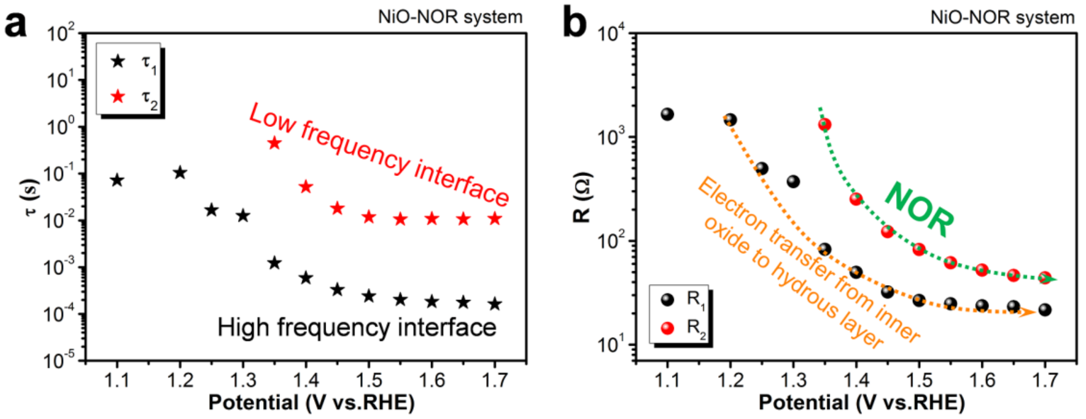

Figure S10. (a) Time constants (τ_1 and τ_2) versus potential for NiO electrode in the NOR system. (b) Equivalent resistances (R_1 and R_2) versus potential for NiO electrode in the NOR system.

For the NiO electrode in the NOR system, the equivalent resistance of R_2 (corresponding to NOR) decreases at a potential above 1.35 V; hence, NOR takes place at the low frequency interface between surface hydrous layer and EDL. Based on the OER mechanism, OER intermediates are generated at the low frequency interface [7-9].

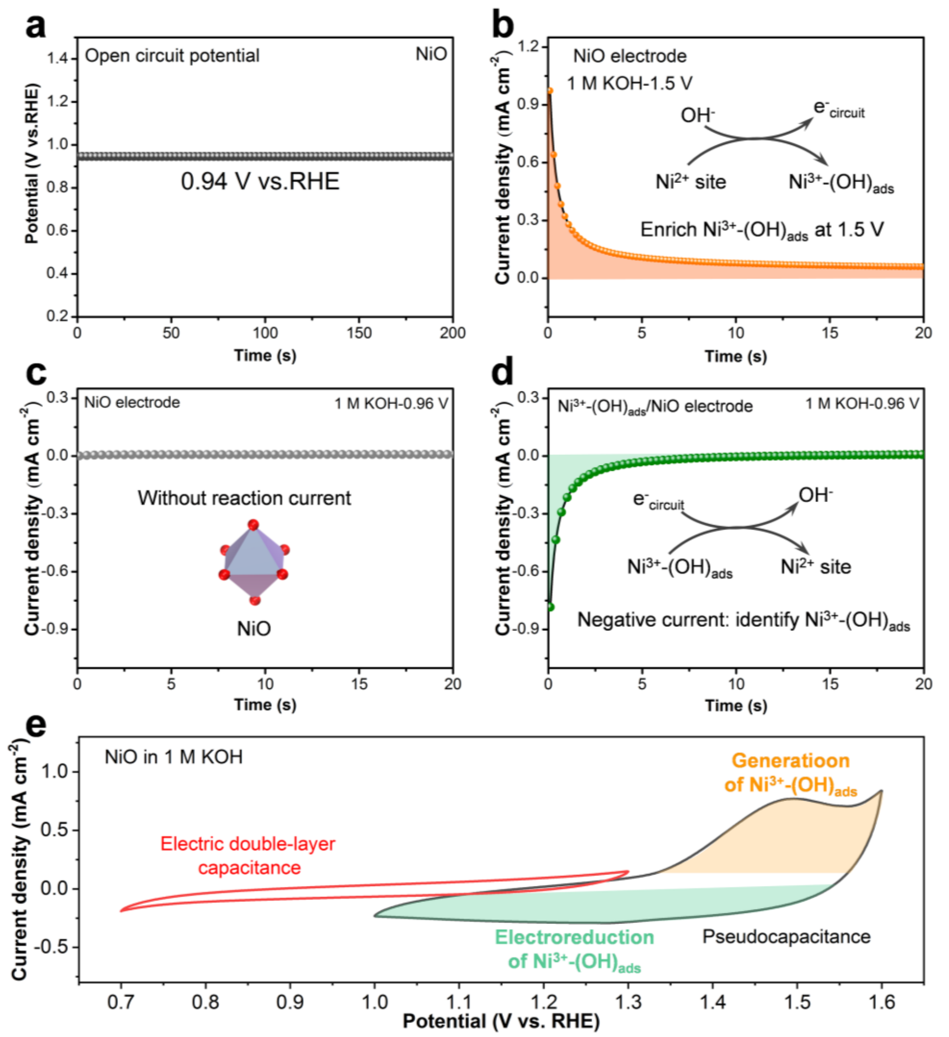

Figure S11. (a) Open circuit potential of the NiO electrode in 1 M KOH. (b) Current density versus time for the NiO electrode at 1.5 V in 1 M KOH. (c, d) Current density versus time for the NiO (c) and Ni^{3+}-$(OH)_{ads}$/NiO (d) electrodes at 0.94 V in 1 M KOH. (e) CV curves of the NiO electrode in 1 M KOH with the scan rate of 50 mV s^{-1}.

1.50 V was adopted to enrich Ni^{3+}-$(OH)_{ads}$ on the NiO electrode; the positive current is corresponding to the generation of Ni^{3+}-$(OH)_{ads}$ (Ni^{2+} + OH^{-} = Ni^{3+}-$(OH)_{ads}$ + $e^{-}_{circuit}$). 0.94 V was adopted to identify Ni^{2+} site and Ni^{3+}-$(OH)_{ads}$: (1) No negative current indicates that there is only Ni^{2+} site, without Ni^{3+}-$(OH)_{ads}$; (2) Negative current is corresponding to the electroreduction of Ni^{3+}-$(OH)_{ads}$ (Ni^{3+}-$(OH)_{ads}$ + $e^{-}_{circuit}$ = Ni^{2+} + OH^{-}) [10-12]. To identify the effect of simply capacitive current, we have supplemented cyclic voltammetry study. Because the electrochemical generation of Ni^{3+}-$(OH)_{ads}$ takes place at a potential above 1.35 V, we carry out the potential window of 0.7 to 1.3 V and the potential window of 1.1 to 1.7 V to identify the electric double-layer capacitance (*i.e.*, simply capacitive current) and the sum of electric double-layer capacitance and pseudocapacitance, respectively. As shown in Fig. S11e, the pseudocapacitance (related to Ni^{3+}-$(OH)_{ads}$) far outweighs the electric double-layer capacitance (*i.e.*, simply capacitive current). Consequently, the negative current (Fig. S11d) is linked mainly to the electroreduction of Ni^{3+}-$(OH)_{ads}$, rather than the simply capacitive current.

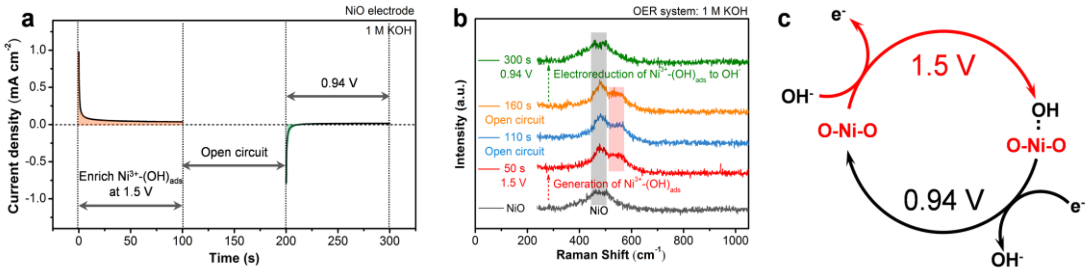

Figure S12. (a) Current density versus time for NiO electrode (0 to 100 s: 1.5 V; 100 to 200 s: open-circuit condition; 200 to 300 s: 0.94 V) in 1 M KOH (b) Synchronous *in situ* Raman spectra for the NiO electrode in the electrochemical testing. (c) Corresponding schematic diagram showing the electrocatalyst function of NiO in the OER system.

For the NiO electrode in 1 M KOH, Ni^{3+}-$(OH)_{ads}$ was generated and accumulated at 1.50 V (0 to 100 s); the generated Ni^{3+}-$(OH)_{ads}$ still existed under open-circuit condition (100 to 200 s); the generated Ni^{3+}-$(OH)_{ads}$ disappeared completely at 0.94 V (200 to 300 s) because of the electroreduction of Ni^{3+}-$(OH)_{ads}$.

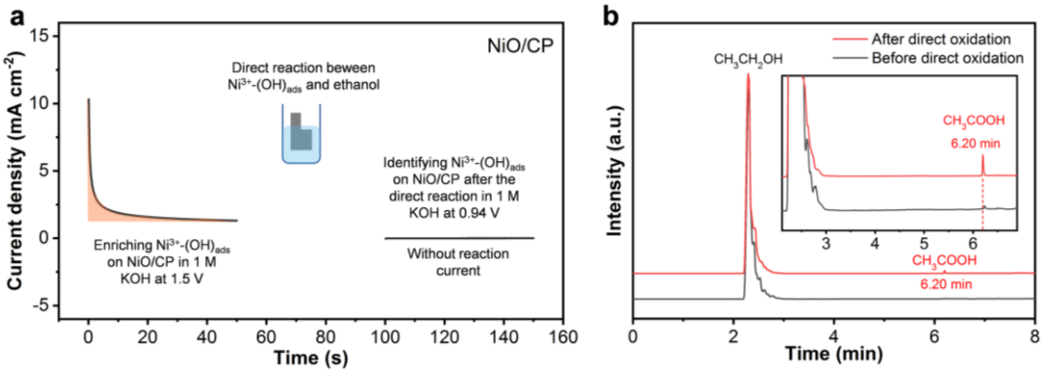

Figure S13. (a) Current density versus time for NiO/CP electrode (0 to 50 s: 1.5 V; 100 to 150 s: 0.94 V) in 1 M KOH. At 50 to 100 s, the NiO/CP containing abound Ni^{3+}-$(OH)_{ads}$ were transferred into the solution (5 mL KOH with 50 mM ethanol), and reacted with ethanol. (b) GC spectra of the solution containing ethanol before/after the reaction between Ni^{3+}-$(OH)_{ads}$ and ethanol (tenfold cycles).

To increase the concentration of carboxylic acid products, we carried out carbon paper electrode (1 cm^2), increased cycle number (tenfold cycles), decreased the volume of the electrolyte containing nucleophile for the verification experiment. Firstly, the controlled potential electrolysis of NiO loading on carbon paper (NiO/CP; 1 cm^2) in 1 M KOH was run at 1.5 V until the charge stopped changing significantly, typically after about 50 s. After charging, abundant Ni^{3+}-$(OH)_{ads}$ intermediates were accumulated on the NiO/CP electrode. Secondly, the NiO/CP electrode containing Ni^{3+}-$(OH)_{ads}$ was removed from the electrolyte of 1 M KOH and then transferred to the solution (5mL; 1 M KOH with 50 mM ethanol) to oxidize ethanol. Thirdly, after a sufficient reaction between Ni^{3+}-$(OH)_{ads}$ and ethanol, the NiO/CP electrode was rinsed with deionized water and then transferred to the electrolyte (1 M KOH). There are almost no reduction current for the electroreduction of Ni^{3+}-$(OH)_{ads}$ at 0.94 V, suggesting that, Ni^{3+}-$(OH)_{ads}$ were depleted on the NiO/CP electrode after reacting with ethanol. We repeated the cycle described above ten times to increase the concentration of carboxylic acid product in the solution. As shown in Fig. 13b, a small number of acetic acid was generated in the solution after the reaction between Ni^{3+}-$(OH)_{ads}$ and ethanol (tenfold cycles). This result proves that Ni^{3+}-$(OH)_{ads}$ can catalyze the spontaneous oxidation of ethanol to acetic acid.

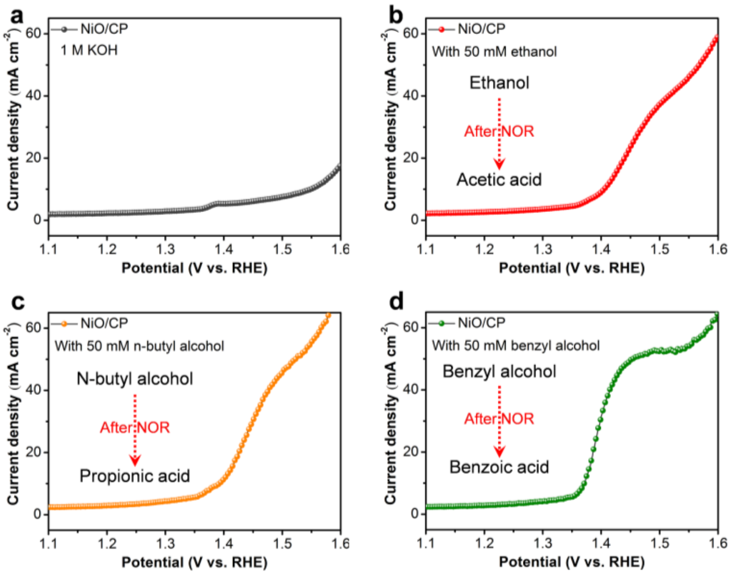

Figure S14. Anodic polarization curves of the NiO/CP electrode in OER system (a) and NOR systems containing ethanol (b), n-propanol (c), and benzyl alcohol (d). In order to increase the electrolysis efficiency, NiO was painted on carbon paper (CP), defined as NiO/CP.

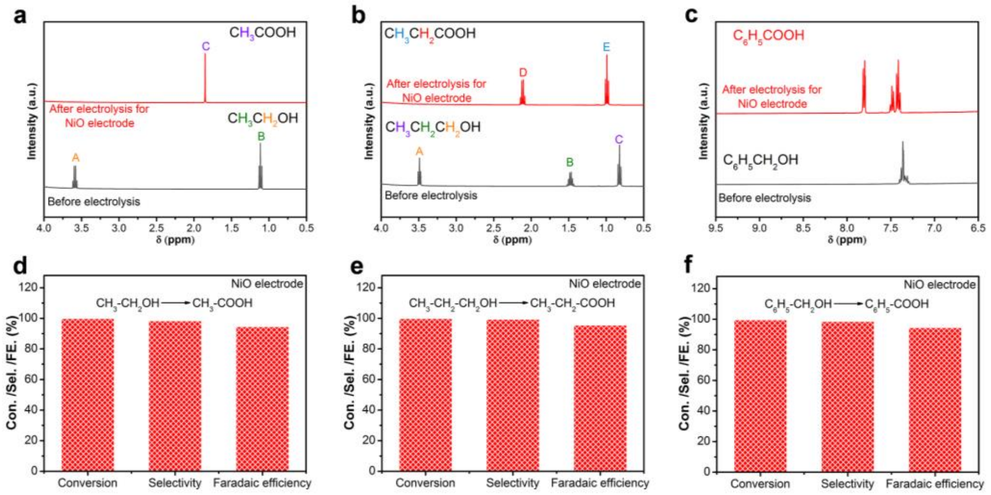

Figure S15. (a-c) 1H nuclear magnetic resonance (1H NMR) spectra for electrolytes before and after electrolysis (1 M KOH with 50 mM R-CH_2OH) for the electrooxidations of ethanol (a), n-butyl alcohol (b), and benzyl alcohol (c) on NiO. (d-f) Conversion rates, selectivities, and Faradaic efficiencies for the electrooxidations of ethanol (d), n-butyl alcohol (e), and benzyl alcohol (f) on NiO.

For the NiO electrode, the electrooxidation products of ethanol, n-butyl alcohol, and benzyl alcohol are acetic acid, propionic acid, and benzoic acid, respectively. Based on gas chromatography mass spectrometry (GC-MS), the conversion rates of different primary alcohols and the selectivities of different electrooxidation products are close to ~100%. Due to the low electrolytic efficiency for the NOR system with a low concentration of nucleophile, Faradaic efficiencies are relatively low, yet above 91%. All in all, in NOR system for NiO, the electrooxidation product of R-CH_2OH is R-COOH.

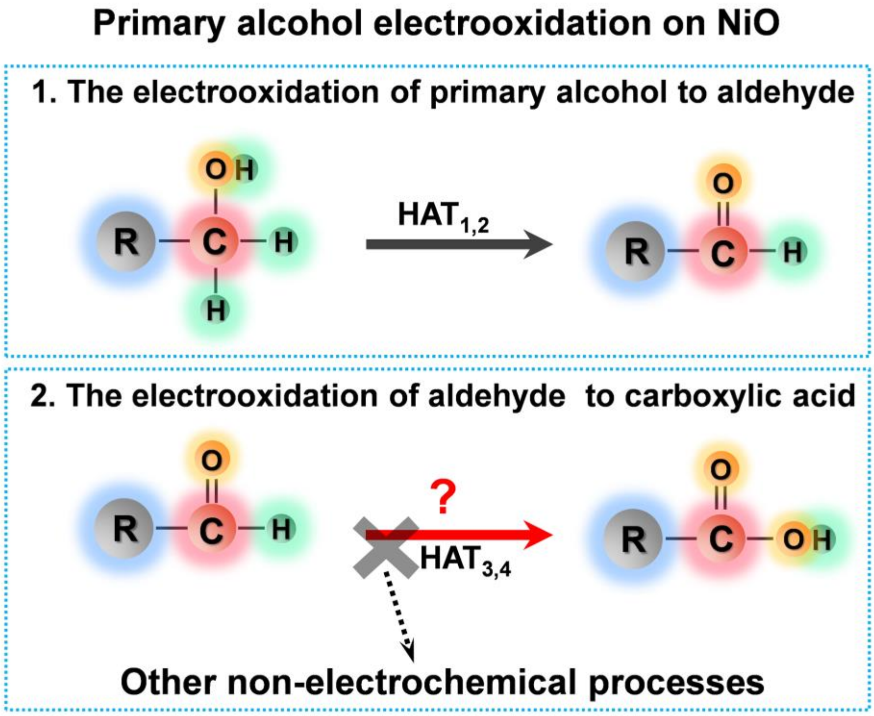

Figure S16. Limitation of EOM involving HAT on the electrooxidation of R-CH_2OH to R-COOH.

The electrooxidation of R-CH_2OH to R-COOH can be divided into two parts, including the electrooxidation of R-CH_2OH to R-CHO and the electrooxidation of R-CHO to R-COOH. In NOR system, EOM involving HAT includes the electrochemical generation of Ni^{3+}-$(OH)_{ads}$ and the Ni^{3+}-$(OH)_{ads}$-induced HAT. In NOR system for NiO, the electrochemical step is not directly related to nucleophile, and the non-electrochemical step is directly related to nucleophile; hence, the nucleophile oxidation pathway depends on the non-electrochemical step, instead of the electrochemical step. The oxidation of R-CH_2OH to R-CHO can be explained by EOM involving HAT: through two Ni^{3+}-$(OH)_{ads}$-induced HAT steps, R-CH_2OH loses two protons and two electrons to generate R-CHO. However, for the oxidation of R-CHO to R-COOH, R-CHO loses two electrons and gets one oxygen atom, which cannot be explained by EOM involving HAT. Hence, in addition to Ni^{3+}-$(OH)_{ads}$-induced HAT, there must be other non-electrochemical processes for the electrooxidation of R-CHO to R-COOH.

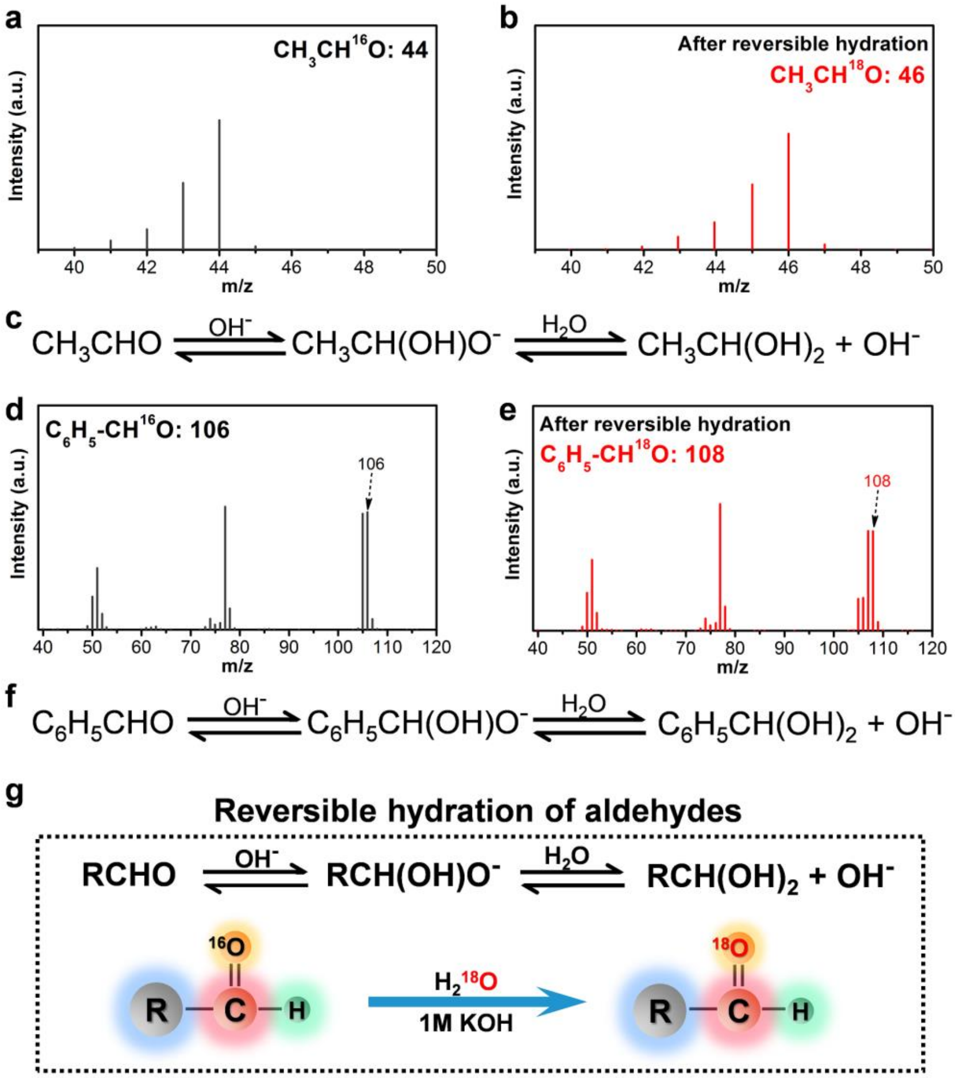

Figure S17. (a, d) Mass spectra of CH_3-$CH^{16}O$ (a) and C_6H_5-$CH^{16}O$ (d) in 1 M KOH $H_2{}^{16}O$ solution. (b, e) Mass spectra of CH_3-$CH^{18}O$ (b) and C_6H_5-$CH^{18}O$ (e) after the reversible hydration (~5 min) in 1 M KOH $H_2{}^{18}O$ solution. (c, f and g) Schematic diagrams showing the reversible hydrations of CH_3-CHO (c), C_6H_5-CHO (f), and R-CHO (g).

To verify the presence of R-$CH(OH)_2$ in the NOR system (1 M KOH aqueous solution containing nucleophile), the ^{18}O isotope tracing experiment was carried out. The relative molecular mass of commercial acetaldehyde is 44 (CH_3-$CH^{16}O$). For the commercial acetaldehyde dissolving in 1 M KOH $H_2{}^{18}O$ solution for ~5 minutes, the relative molecular mass of acetaldehyde is 46, corresponding to CH_3-$CH^{18}O$. It proves that almost all of ^{16}O atoms in CH_3-CHO were replaced by the ^{18}O atom in $H_2{}^{18}O$. The same is true for benzaldehyde (C_6H_5-CHO). Almost all of ^{16}O atoms in C_6H_5-CHO were replaced by the ^{18}O atom in 1 M KOH $H_2{}^{18}O$ solution within ~5 minutes as well. Hence, in the NOR system, the hydration of R-CHO to R-$C(OH)_2$ is spontaneous and reversible.

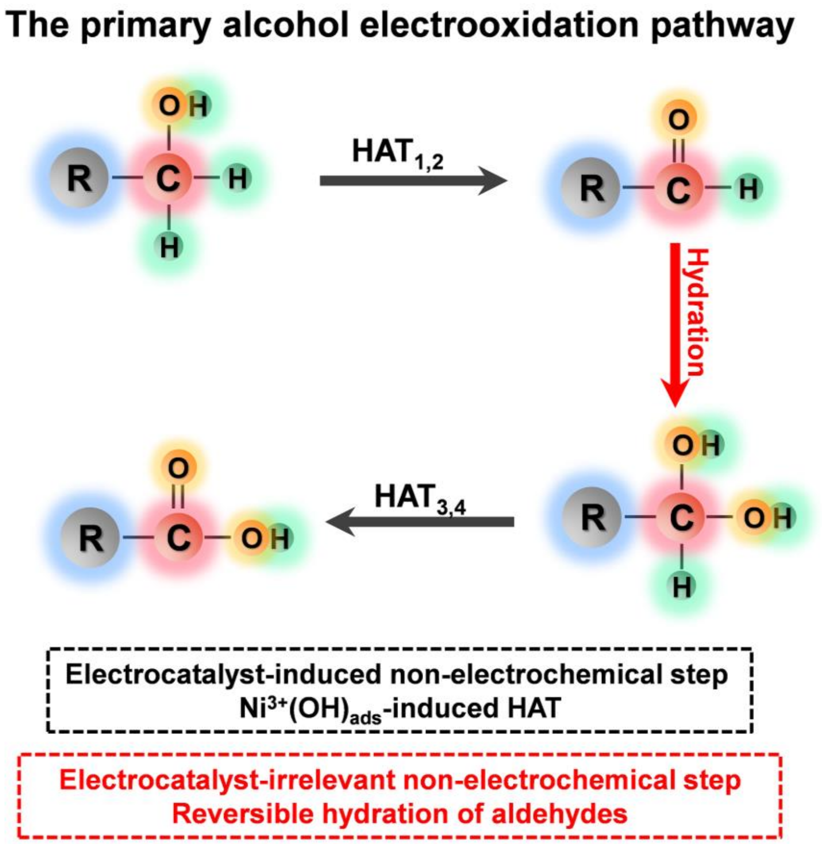

Figure S18. Schematic diagram showing the primary alcohol oxidation pathway in the NOR system based on NiO.

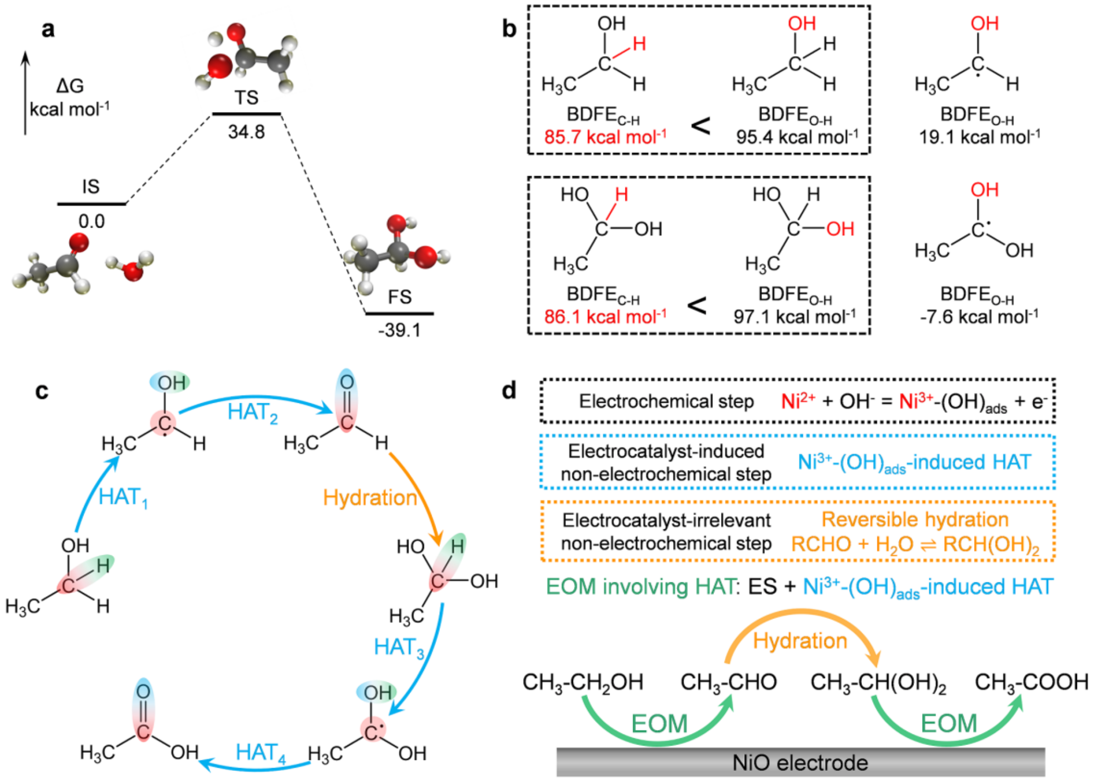

Figure S19. (a) Free energy reaction profile for the hydration of acetaldehyde (CH_3-CHO). (b) BDFEs of X-H bonds in CH_3-CH_2OH, CH_3-·CHOH, CH_3-$CH(OH)_2$, and CH_3-·$C(OH)_2$. (c) Reaction pathway for the electrooxidation of CH_3-CH_2OH to CH_3-COOH based on non-electrochemical steps. (d) Schematic diagram showing the CH_3-CH_2OH electrooxidation on NiO.

In the NOR system, the non-electrochemical step must be spontaneous, and identifying the reaction sequence of different non-electrochemical steps is vital to understand the NOR pathway. For the oxidation of CH_3-CH_2OH to CH_3-COOH, four Ni^{3+}-$(OH)_{ads}$-induced HAT steps and one hydration step are involved. Through transition states (TS) calculation, the hydration of CH_3-CHO only needs to overcome low barrier of TS (34.8 kcal mol^{-1}), and the free energy of final state (FS) is -39.1 kcal mol^{-1}. The DFT calculation also supports the spontaneous hydration of CH_3-CHO to CH_3-$CH(OH)_2$. It is difficult to identify the reaction sequence of HAT steps in the alcohols electrooxidation through experiment. The HAT step is essentially the X-H bond cleavage in nucleophile. The bond dissociation free energy (BDFE) can describe the free energy of the X-H bond cleavage [13, 14]. According to the DFT calculation of CH_3-CH_2OH, BDFEs of the alpha-C-H bond and the O-H bond are 85.7 and 95.4 kcal mol^{-1}, respectively. For CH_3-$CH(OH)_2$, BDFEs of the alpha-C-H bond and the O-H bond are 86.1 and 97.1 kcal mol^{-1}, respectively. Hence, the dehydrogenation of alpha-C-H bond in CH_3-CH_2OH or CH_3-$CH(OH)_2$ takes precedence over the dehydrogenation of O-H bond in CH_3-CH_2OH or CH_3-$CH(OH)_2$. Consequently, we proposed the oxidation pathway for CH_3-CH_2OH to CH_3-COOH, comprising five non-electrochemical steps: (1) dehydrogenation of CH_3-CH_2OH to CH_3-·CHOH (HAT_1), (2) dehydrogenation of CH_3-·CHOH to CH_3-CHO (HAT_2), (3) hydration of CH_3-CHO to CH_3-$CH(OH)_2$, (4) dehydrogenation of CH_3-$CH(OH)_2$ to CH_3-·$C(OH)_2$ (HAT_3), and (5) dehydrogenation of CH_3-·$C(OH)_2$ to CH_3-COOH (HAT_4).

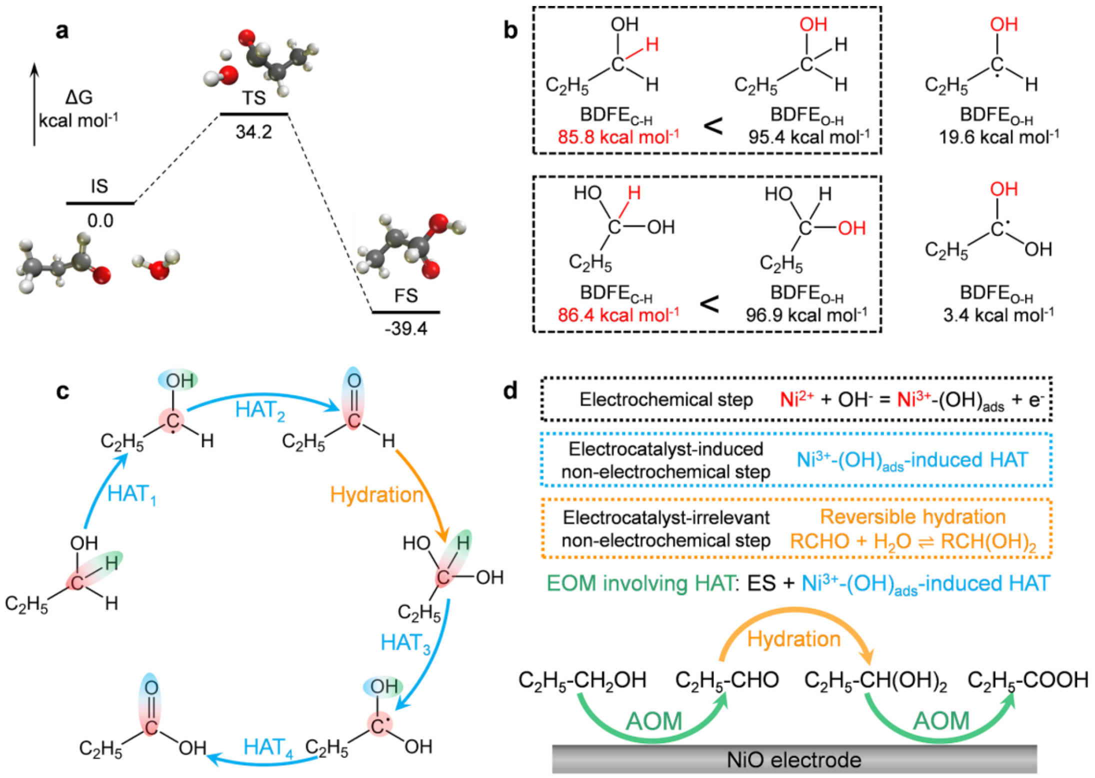

Figure S20. (a) Free energy reaction profile for the hydration of propionaldehyde (CH_3-CH_3-CHO). (b) BDFEs of X-H bonds in CH_3-CH_2-CH_2OH, CH_3-CH_2-·CHOH, CH_3-CH_2-$CH(OH)_2$, and CH_3-CH_2-·$C(OH)_2$. (c) Reaction pathway of the electrooxidation of CH_3-CH_2-CH_2OH to CH_3-CH_2-COOH based on non-electrochemical steps. (d) Schematic diagram showing the CH_3-CH_2-CH_2OH electrooxidation on NiO.

For the CH_3-CH_2-CH_2OH electrooxidation reaction, the result is highly similar. Through TS calculation, the hydration of CH_3-CH_3-CHO only needs to overcome low barrier of TS (34.2 kcal mol^{-1}), and the free energy of final state (FS) is -39.4 kcal mol^{-1}. The DFT calculation also supports the spontaneous hydration of CH_3-CH_3-CHO to CH_3-CH_3-$CH(OH)_2$. According to the BDFEs, the dehydrogenation of alpha-C-H bond in CH_3-CH_2-CH_2OH or CH_3-CH_2-$CH(OH)_2$ takes precedence over the dehydrogenation of O-H bond in CH_3-CH_2-CH_2OH or CH_3-CH_2-$CH(OH)_2$. Consequently, we proposed the oxidation pathway for CH_3-CH_2-CH_2OH to CH_3-CH_2-COOH, comprising five non-electrochemical steps: (1) dehydrogenation of CH_3-CH_2-CH_2OH to CH_3-CH_2-·CHOH (HAT_1), (2) dehydrogenation of CH_3-CH_2-·CHOH to CH_3-CH_2-CHO (HAT_2), (3) hydration of CH_3-CH_2-CHO to CH_3-CH_2-$CH(OH)_2$, (4) dehydrogenation of CH_3-CH_2-$CH(OH)_2$ to CH_3-CH_2-·$C(OH)_2$ (HAT_3), and (5) dehydrogenation of CH_3-CH_2-·$C(OH)_2$ to CH_3-CH_2-COOH (HAT_4).

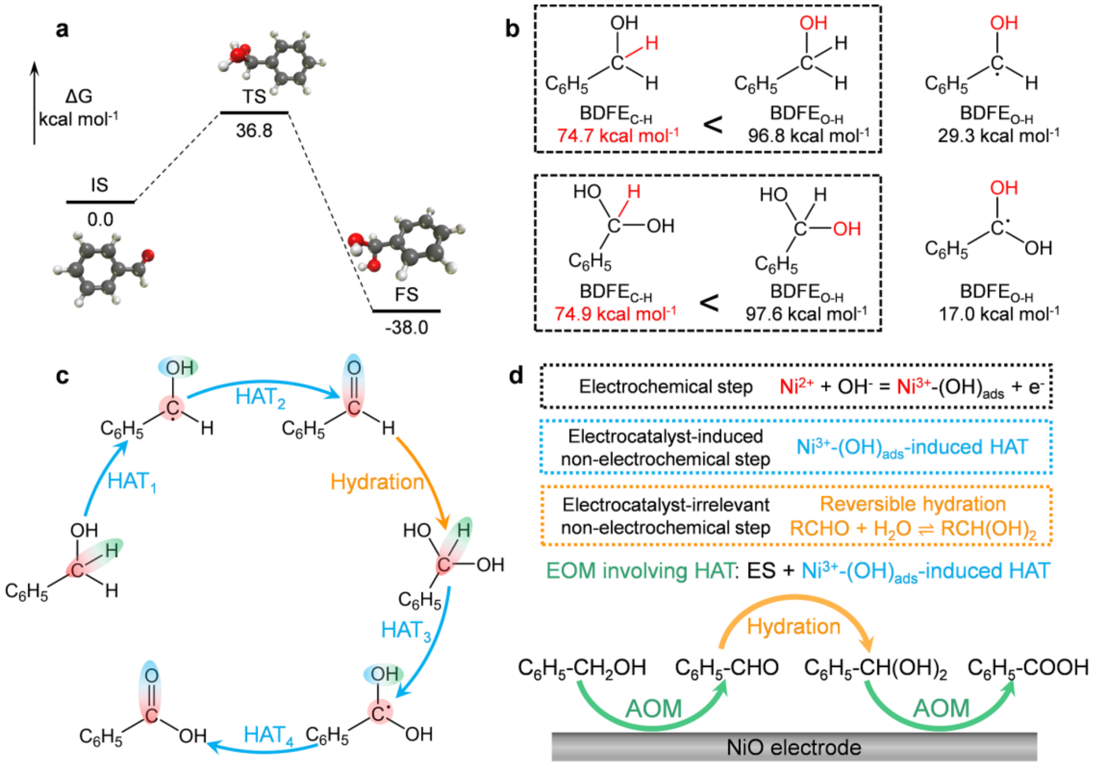

Figure S21. (a) Free energy reaction profile for the hydration of C_6H_5-CHO. (b) BDFEs of X-H bonds in C_6H_5-CH_2OH, C_6H_5-·CHOH, C_6H_5-CH$(OH)_2$, and C_6H_5-·C$(OH)_2$. (c) Reaction pathway of the electrooxidation of C_6H_5-CH_2OH to C_6H_5-COOH based on non-electrochemical steps. (d) Schematic diagram showing the C_6H_5-CH_2OH electrooxidation on NiO.

For the primary alcohols electrooxidation reactions, the results are highly similar. The BDFE of alpha-C-H bond in R-CH_2OH or R-CH$(OH)_2$ (R:CH_3-, CH_3-CH_2- and C_6H_5-) is less than the BDFE of O-H bond in R-CH_2OH or R-CH$(OH)_2$. Hence, the dehydrogenation of alpha-C-H bond in R-CH_2OHR-CH_2OH or R-CH$(OH)_2$ takes precedence over the dehydrogenation of O-H bond. All in all, on the NiO electrode, R-CH_2OH can be electrochemically oxidized to R-COOH due to the synergy of EOM involving HAT and the hydration of R-CHO. The oxidation pathway for R-CH_2OH to R-COOH comprises five non-electrochemical steps: (1) dehydrogenation of R-CH_2OH to R-·CHOH (HAT_1), (2) dehydrogenation of R-·CHOH to R-CHO (HAT_2), (3) hydration of R-CHO to R-CH$(OH)_2$, (4) dehydrogenation of R-CH$(OH)_2$ to R-·C$(OH)_2$ (HAT_3), and (5) dehydrogenation of R-·C$(OH)_2$ to R-COOH (HAT_4).

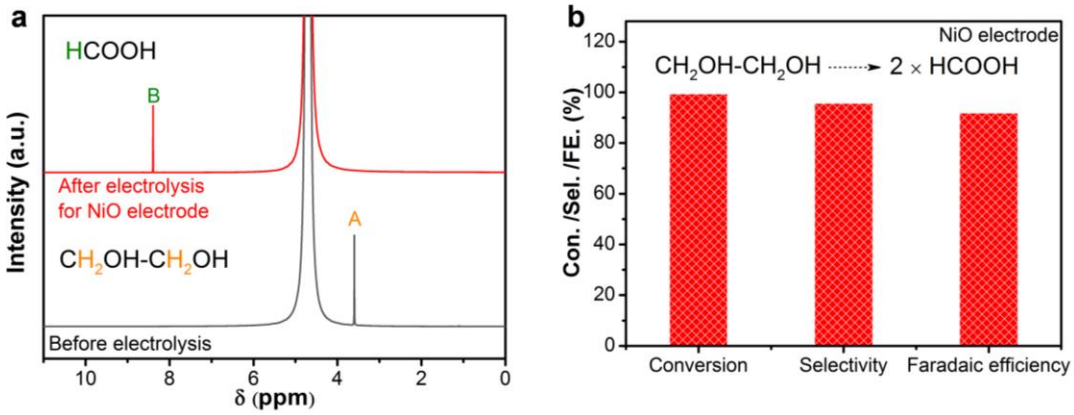

Figure S22. (a) 1H NMR spectra for electrolytes before and after electrolysis (1 M KOH with 50 mM CH_2OH-CH_2OH) for the electrooxidation of CH_2OH-CH_2OH. (b) Conversion rate, selectivity, and Faradaic efficiency for the electrooxidation of CH_2OH-CH_2OH on the NiO electrode.

For the NiO electrode, the electrooxidation product of CH_2OH-CH_2OH is HCOOH. Based on high performance liquid chromatography (HPLC), one CH_2OH-CH_2OH molecule can be electrochemically oxidized to two HCOOH molecules on the NiO electrode, accompanied with the C-C bond cleavage. The conversion rate, selectivity, and Faradaic efficiency for the electrooxidation of CH_2OH-CH_2OH are 99.4%, 95.6%, and 91.8%, respectively.

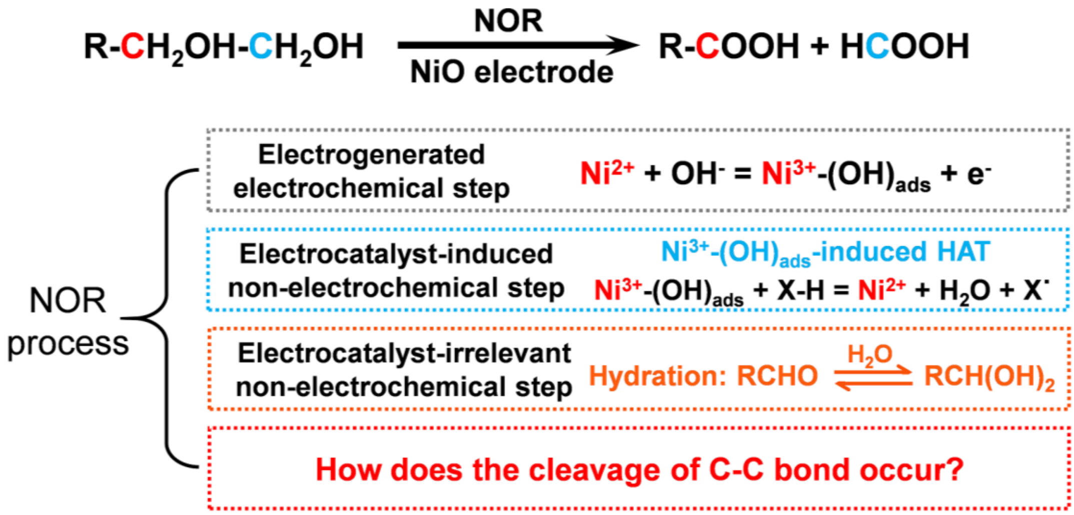

Figure S23. Schematic diagram showing the basic steps in the electrooxidation of R-CHOH-CH_2OH on the NiO electrode, including the electrochemical generation of Ni^{3+}-$(OH)_{ads}$, the Ni^{3+}-$(OH)_{ads}$-induced HAT, the reversible hydration of aldehyde, and the cleavage of C-C bond.

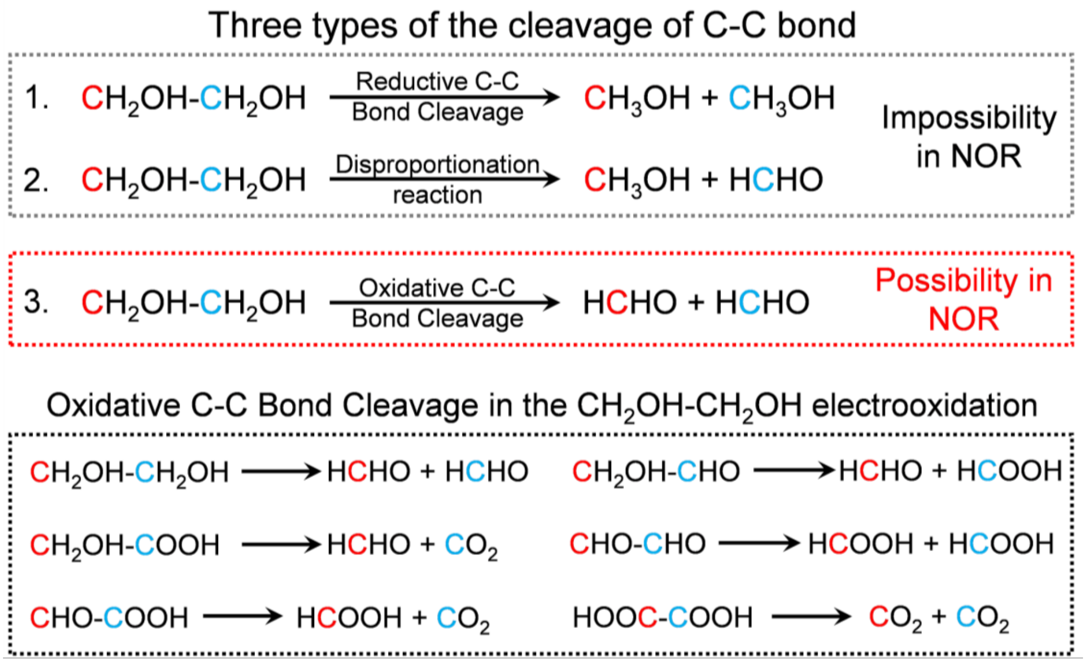

Figure S24. Three types of the cleavage of C-C bond, including reduction, disproportionation, and oxidation reactions.

Take the C-C bond cleavage of CH_2OH-CH_2OH for instance. For the reductive C-C bond cleavage of CH_2OH-CH_2OH, the product is methyl alcohol (CH_3OH). For the disproportionation reaction of C-C bond cleavage of CH_2OH-CH_2OH, one hydroxymethyl group is oxidized to formaldehyde (HCHO), and the other one is reduced to CH_3OH. For the oxidative C-C bond cleavage of CH_2OH-CH_2OH, the product is HCHO. Because NOR is an oxidation half reaction, non-spontaneous reduction reaction cannot happen in the NOR system under the oxidation potential. The reduction of hydroxymethyl group to CH_3OH is a non-spontaneous process, which indicates that the reductive C-C bond cleavage and the disproportionation reaction of C-C bond cleavage are impossible to happen in the NOR system. Evidently the only possibility is the oxidative C-C bond cleavage in the NOR system [15].

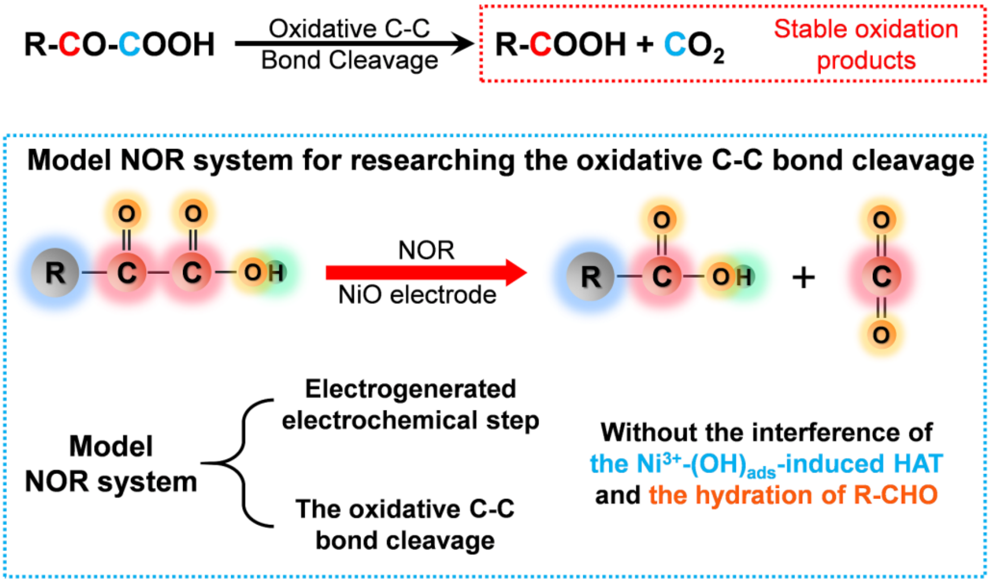

Figure S25. Schematic diagram showing the model NOR system (the electrooxidation of R-CO-COOH) for researching the oxidative C-C bond cleavage on NiO.

The electrochemical generation of Ni^{3+}-$(OH)_{ads}$ (electrochemical step) is unavoidable for NOR of NiO. Therefore, as for the ideal model reaction system for researching the oxidative C-C bond cleavage, two non-electrochemical steps *i.e.*, the Ni^{3+}-$(OH)_{ads}$-induced HAT and the hydration of R-CHO, should be avoided. Theoretically, the alpha ketonic acid (R-CO-COOH) can be electrochemically oxidized to R-COOH and CO_2, containing only the oxidative C-C bond cleavage process [16, 17]. Hence, the R-CO-COOH electrooxidation is an ideal model for researching the oxidative C-C bond cleavage on the NiO electrode.

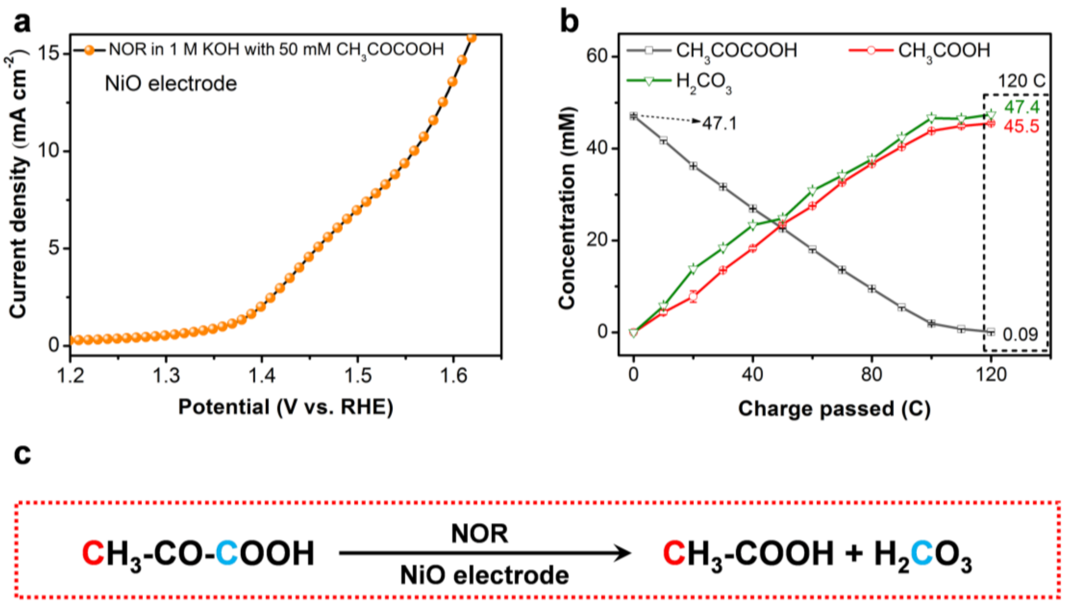

Figure S26. (a) Anodic polarization curve of NiO electrode in 1 M KOH with 50 mM CH_3-CO-COOH. (b) Concentration changes of CH_3-CO-COOH and its oxidation products (CH_3COOH and H_2CO_3) versus charge passed for the electrooxidation of CH_3-CO-COOH on the NiO electrode. (c) The electrooxidation of CH_3-CO-COOH to CH_3COOH and H_2CO_3 on the NiO electrode.

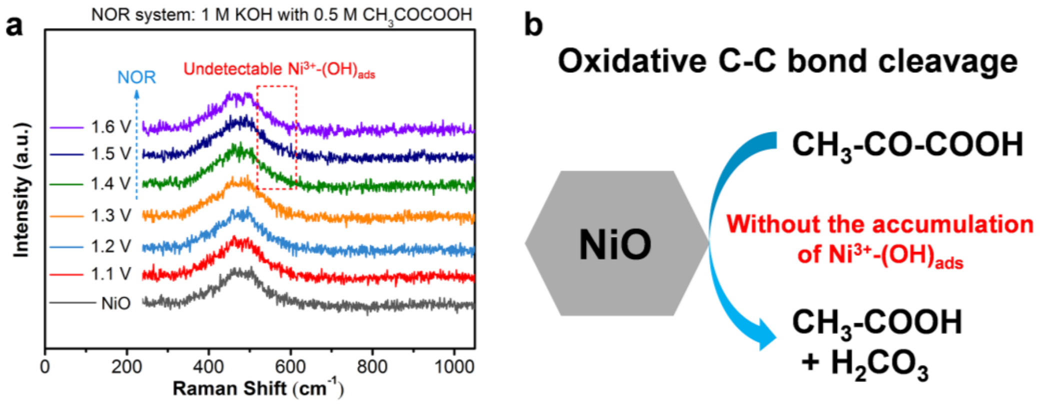

Figure S27. (a) *In situ* Raman spectra of NiO electrode in 1 M KOH with 0.5 M CH_3-CO-COOH. (b) Schematic illustration of NiO electrode surface species evolution during the electrooxidation of CH_3-CO-COOH.

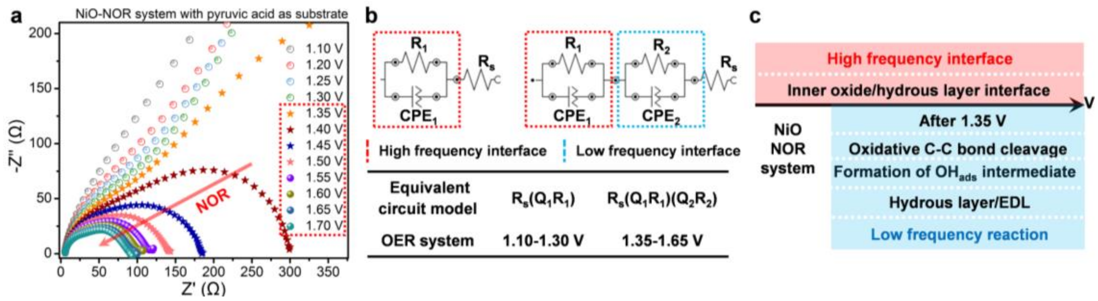

Figure S28. Nyquist (a), equivalent circuit models (b), and potential-dependent behavior (c) for the oxidative C-C bond cleavage of CH_3-CO-COOH on the NiO electrode (1 M KOH with 0.5 M CH_3-CO-COOH).

The corresponding EIS-fitting result is listed in Supplementary Table 3. In the NOR system containing CH_3-CO-COOH for NiO electrode, there is only one semicircle at 1.10 to 1.30 V at Nyquist plots, and there are two semicircles at a potential above 1.35 V at Nyquist plots. Therefore, $R_s(Q_1R_1)$ and $R_s(Q_1R_1)(Q_2R_2)$ models were employed for the electrooxidation of CH_3-CO-COOH on NiO at the potential range of 1.10 to 1.30 V and the potential range of 1.35 to 1.70 V, respectively. In the NOR system containing CH_3-CO-COOH for NiO electrode, the electrochemically induced oxidative C-C bond cleavage of CH_3-CO-COOH takes place at a potential above ~1.35 V.

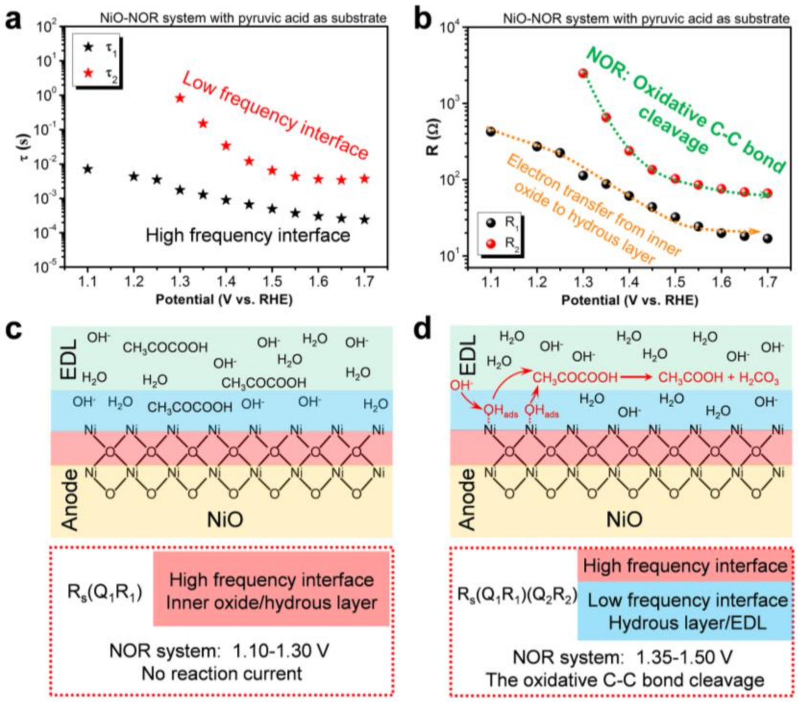

Figure S29. (a) Time constants (τ_1 and τ_2) versus potential for the electrooxidation of CH_3-CO-COOH on NiO. (b) Equivalent resistances (R_1 and R_2) versus potential for the electrooxidation of CH_3-CO-COOH on NiO. (c, d) Schematic diagrams showing the NOR system containing CH_3-CO-COOH of NiO electrode at different potential ranges.

At a potential above 1.35 V in NOR system containing CH_3-CO-COOH for NiO electrode, Ni^{3+}-$(OH)_{ads}$ is formed at the low frequency interface, and the generated Ni^{3+}-$(OH)_{ads}$ can react with CH_3-CO-COOH, thus leading to the oxidative C-C bond cleavage of CH_3-CO-COOH to CH_3-COOH and HCOOH.

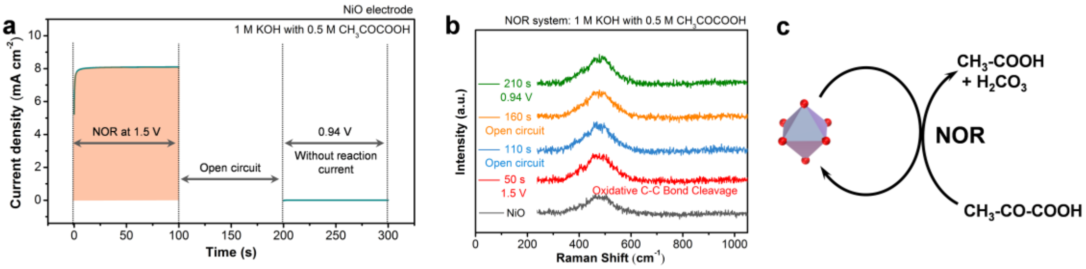

Figure S30. (a, b) Current density versus time (a) and *in situ* Raman spectra (b) for NiO electrode in NOR system containing CH_3-CO-COOH (0 to 100 s: 1.50 V; 100 to 200 s: open circuit state; 200 to 300 s: 0.94 V). (c) Schematic diagram showing the electrooxidation of CH_3-CO-COOH on NiO electrode.

In 1 M KOH with 0.5 M CH_3-CO-COOH, Ni^{3+}-$(OH)_{ads}$ could not be accumulated during the electrochemically induced oxidative C-C bond cleavage at 1.5 V (0 to 100 s), and Ni^{3+}-$(OH)_{ads}$ was not detected under open-circuit condition (100 to 200 s) and at 0.94 V (200 to 300 s). For the CH_3-CO-COOH electrooxidation on the NiO electrode, the electrochemically induced oxidative C-C bond cleavage of CH_3-CO-COOH takes place continuously under the oxidation potential, without the accumulation of Ni^{3+}-$(OH)_{ads}$.

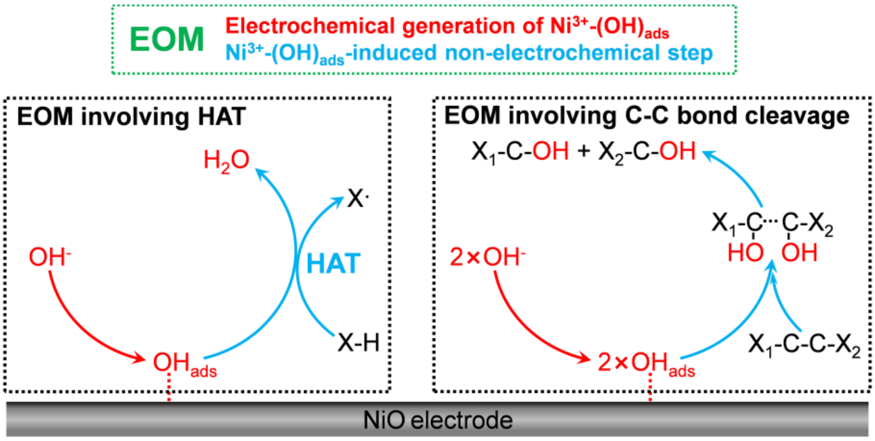

Figure S31. Two EOMs including electrochemical step and electrocatalyst-induced non-electrochemical step: (1) EOM involving HAT including the electrochemical generation of Ni^{3+}-$(OH)_{ads}$ and Ni^{3+}-$(OH)_{ads}$-induced HAT; (2) EOM involving C-C bond cleavage including the electrochemical generation of Ni^{3+}-$(OH)_{ads}$ and Ni^{3+}-$(OH)_{ads}$-induced C-C bond cleavage.

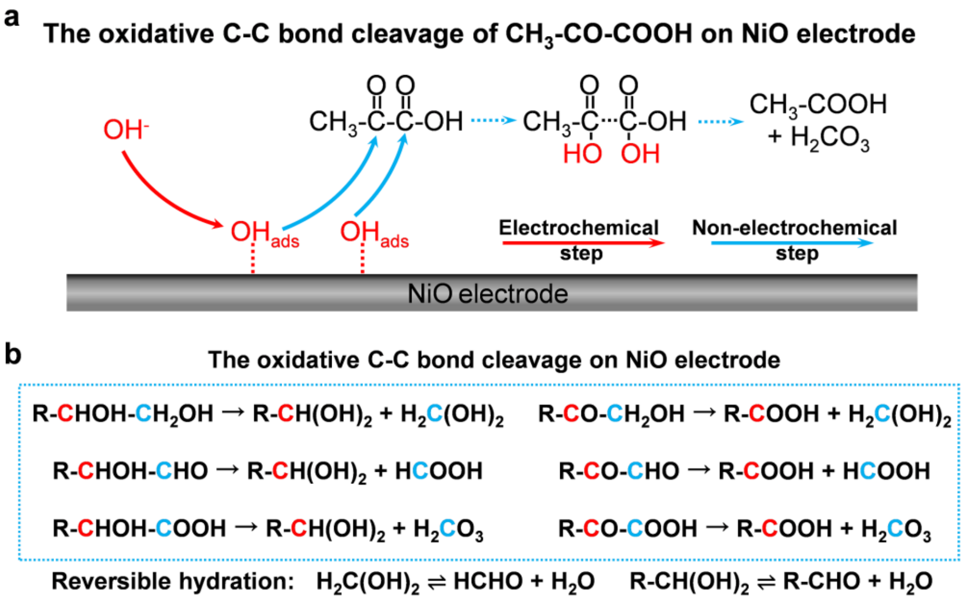

Figure S32. (a) Schematic diagram showing the electrochemically induced oxidative C-C bond cleavage of CH_3-CO-COOH on NiO electrode. (b) The electrochemically induced oxidative C-C bond cleavage of R-CHOH-CH_2OH or its potential oxidation intermediates.

During EOM involving C-C bond cleavage, two Ni^{3+}-$(OH)_{ads}$ intermediates attack the C-C bond in CH_3-CO-COOH to form two C-OH bonds, thus leading to the spontaneous C-C bond cleavage. In other words, for EOM involving C-C bond cleavage, the C-C bond cleavage is accompanied with the formation of two C-OH bonds. According to EOM involving C-C bond cleavage, there are five possible reaction pathways: (1) Hydroxymethyl group (-CH_2OH) is oxidized to formaldehyde hydrate ($H_2C(OH)_2$), instead of HCHO; (2) Aldehyde group (-CHO) is oxidized to HCOOH; (3) Carboxyl group (-COOH) is oxidized to H_2CO_3, instead of CO_2; (4) Secondary hydroxyl group (R-CHOH-) is oxidized to aldehyde hydrate (R-$CH(OH)_2$), instead of R-CHO; (5) Ketone group (R-CO-) is oxidized to R-COOH.

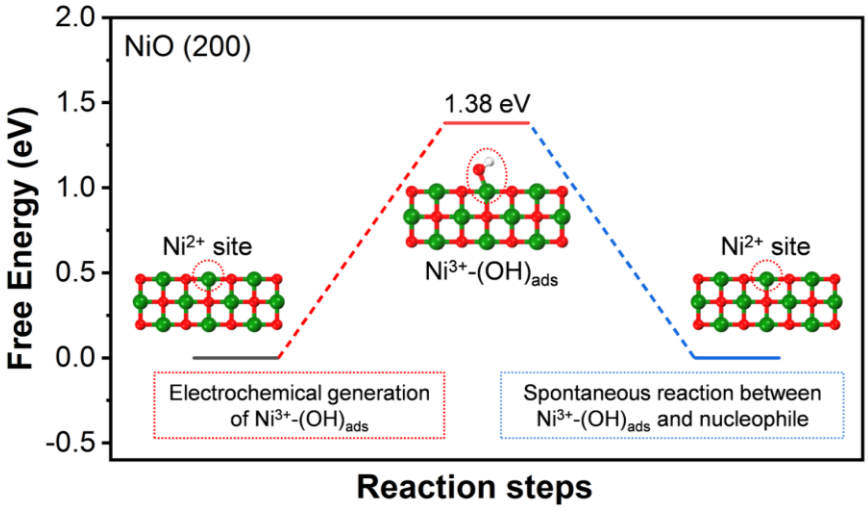

Figure S33. DFT calculation for the electrocatalyst function during the NOR on NiO (200), including the electrochemical generation of Ni^{3+}-$(OH)_{ads}$ and spontaneous non-electrochemical reaction between Ni^{3+}-$(OH)_{ads}$ and nucleophiles.

The free energy change of the generation of Ni^{3+}-$(OH)_{ads}$ is 1.38 eV. Consequently, the electrochemical generation of Ni^{3+}-$(OH)_{ads}$ is the rate-limiting step in the electrocatalyst function during the NOR on NiO.

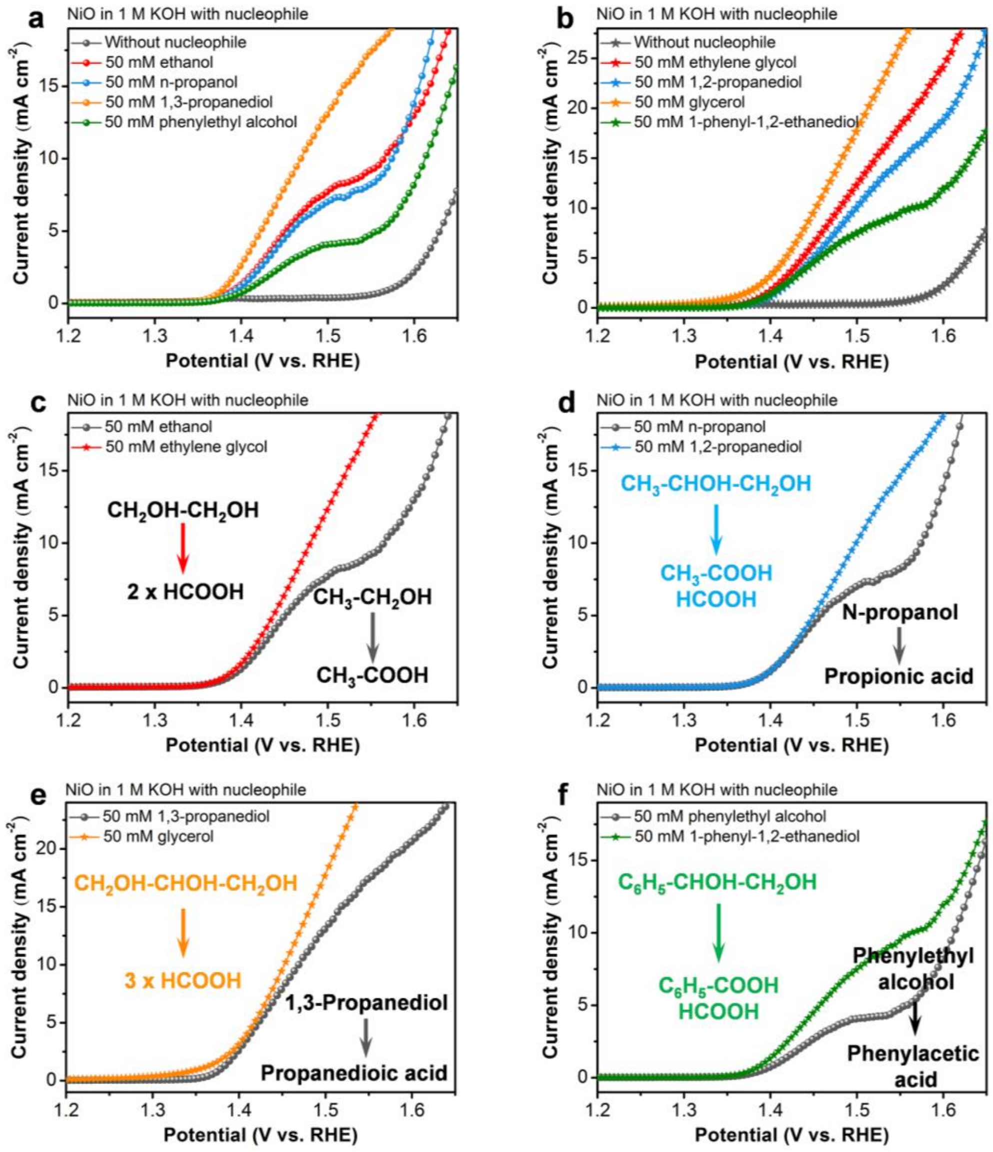

Figure S34. (a, b) Anodic polarization curves of NiO in the NOR system containing R-CH_2OH (such as ethanol, n-propanol, 1,3-propanediol, and phenethyl alcohol) (a) or R-CHOH-CH_2OH (such as ethylene glycol, 1,2-propanediol, glycerol, and 1-phenyl-1,2-ethanediol) (b). (c-f) Comparisons of anodic polarization curves of NiO electrode in different NOR systems containing R-CH_2OH or R-CHOH-CH_2OH.

In the NOR system containing R-CHOH-CH_2OH (such as ethylene glycol, 1,2-propanediol, glycerol, and 1-phenyl-1,2-ethanediol) for NiO electrode, the NOR current is increasing rapidly at a potential above ~1.35 V. The electrochemical performance of the R-CHOH-CH_2OH electrooxidation is superior to that of the R-CH_2OH electrooxidation because R-CHOH-CHOH containing two hydroxyl groups has more reaction sites (*e.g.*, two O-H bonds, two C-H bonds, and one C-C bond) than R-CH_2-CHOH containing one hydroxyl group (reaction site: one C-H bond and one O-H bond).

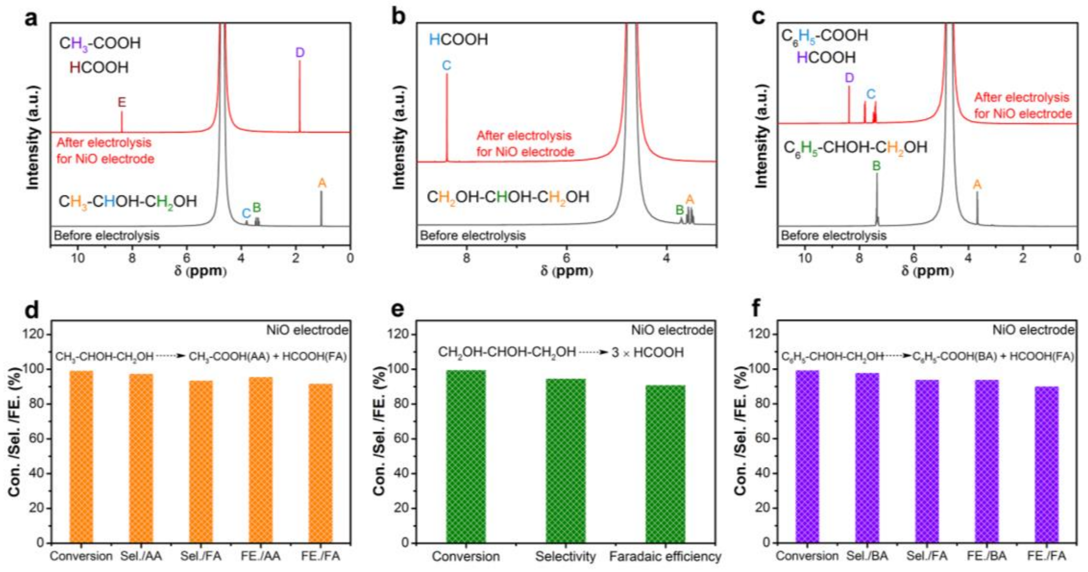

Figure S35. (a-c) 1H NMR spectra for electrolytes before and after electrolysis for the electrooxidations of CH_3-CHOH-CH_2OH (a), CH_2OH-CHOH-CH_2OH (b), and C_6H_5-CHOH-CH_2OH (c) on NiO (1 M KOH with 50 mM nucleophile). (d-f) Conversion rates, selectivities, and Faradaic efficiencies of the electrooxidations of CH_3-CHOH-CH_2OH (d), CH_2OH-CHOH-CH_2OH (e), and C_6H_5-CHOH-CH_2OH (f) on the NiO electrode.

In the NOR system for NiO electrode, one CH_2OH-CH_2OH molecule can be electrochemically oxidized to two HCOOH molecules; one CH_3-CHOH-CH_2OH molecule can be electrochemically oxidized to one CH_3-COOH molecule and one HCOOH molecule; one CH_2OH-CHOH-CH_2OH molecule can be electrochemically oxidized to three HCOOH molecules; one C_6H_5-CHOH-CH_2OH molecule can be electrochemically oxidized to one C_6H_5-COOH molecule and one HCOOH molecule. In summary, on the NiO electrode, one R-CHOH-CH_2OH molecule can be electrochemically oxidized to one R-COOH molecule and one HCOOH molecule, accompanied by the cleavage of C-C bond.

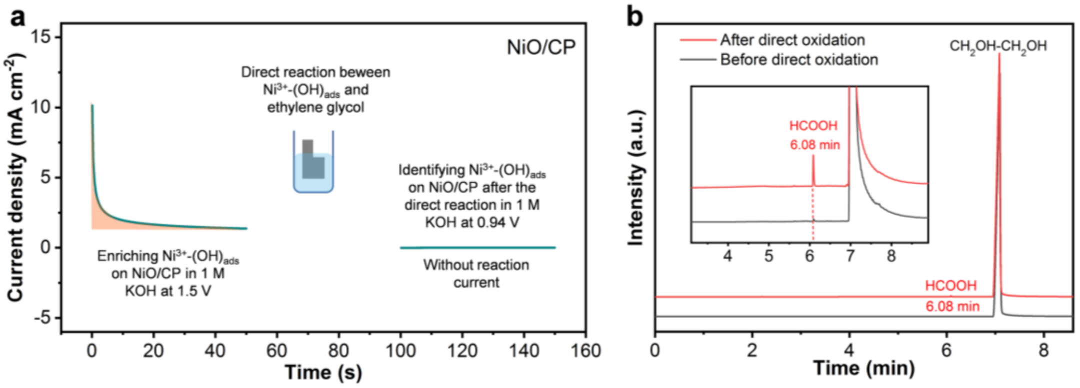

Figure S36. (a) Current density versus time for NiO/CP electrode (0 to 50 s: 1.5 V; 100 to 150 s: 0.94 V) in 1 M KOH. At 50 to 100 s, the NiO/CP containing abound Ni^{3+}-$(OH)_{ads}$ were transferred into the solution (5 mL KOH with 50 mM ethylene glycol) and reacted with ethylene glycol. (b) GC spectra of the solution containing ethylene glycol before/after the reaction between Ni^{3+}-$(OH)_{ads}$ and ethylene glycol (tenfold cycles).

We carried the same experiment as shown in Fig. S13, but with ethylene glycol instead of ethanol as the nucleophile substrate. Firstly, the controlled potential electrolysis of NiO/CP (1 cm^2) in 1 M KOH was run at 1.5 V until the charge stopped changing significantly with time, typically after about 50 s. After charging, abundant Ni^{3+}-$(OH)_{ads}$ intermediates were accumulated on the NiO/CP electrode. Secondly, the NiO/CP electrode containing Ni^{3+}-$(OH)_{ads}$ was removed from the electrolyte of 1 M KOH and then transferred to the solution (5mL; 1 M KOH with 50 mM ethylene glycol) to oxidize ethylene glycol. Thirdly, after a sufficient reaction between Ni^{3+}-$(OH)_{ads}$ and ethylene glycol, the NiO/CP electrode was rinsed with deionized water, and then transferred to the electrolyte (1 M KOH). There are almost no reduction current for the electroreduction of Ni^{3+}-$(OH)_{ads}$ at the potential of 0.94 V, suggesting that, on the NiO/CP electrode, Ni^{3+}-$(OH)_{ads}$ were depleted after the reaction between Ni^{3+}-$(OH)_{ads}$ and ethylene glycol. We repeated the abovementioned cycle ten times to increase the concentration of carboxylic acid product in the solution (5mL; 1 M KOH with 50 mM ethylene glycol). As shown in Fig. 36b, a small number of HCOOH was generated in the solution after the reaction between Ni^{3+}-$(OH)_{ads}$ and ethylene glycol (repeating the cycle ten times). This result proves that Ni^{3+}-$(OH)_{ads}$ can catalyze the spontaneous oxidation of ethylene glycol to HCOOH accompanied by the C-C bond cleavage.

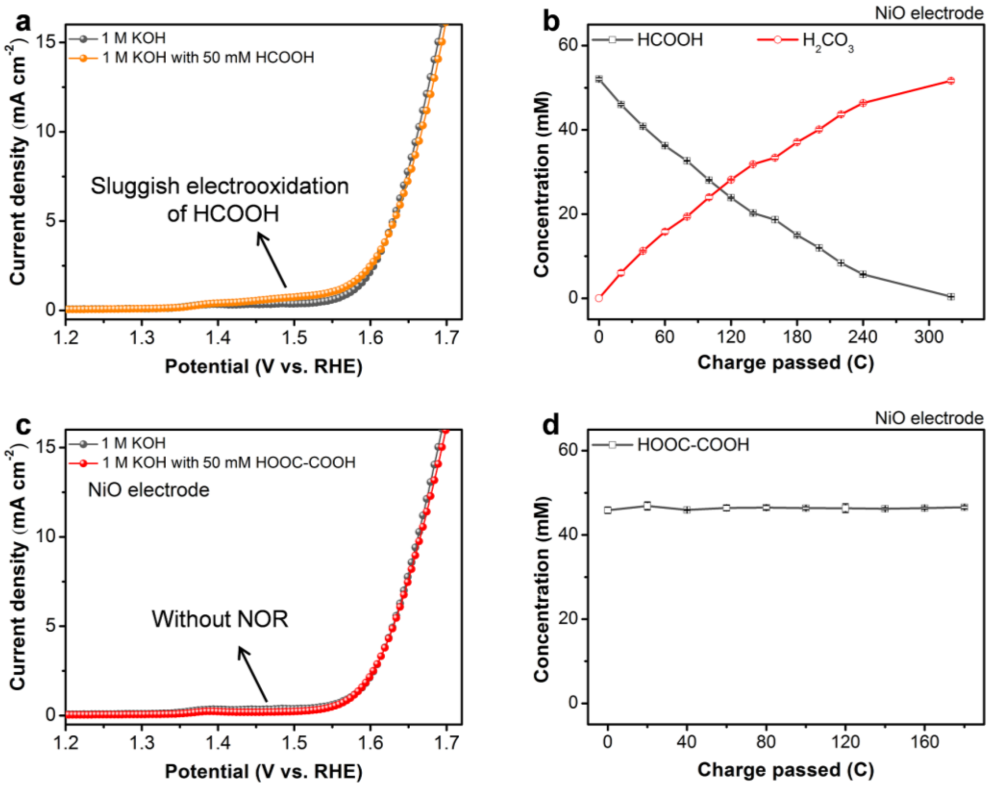

Figure S37. (a, c) Anodic polarization curves of NiO electrode in 1 M KOH with 50 mM HCOOH (a) and in 1 M KOH with 50 mM COOH-COOH (c). (b, d) Concentration changes of reagent and oxidation product for the HCOOH electrooxidation (b) and the HOOC-COOH electrooxidation (d) on the NiO electrode.

We took the CH_2OH-CH_2OH (the simplest vicinal diol) electrooxidation for instance to speculate the reaction mechanism of the R-CHOH-CH_2OH electrooxidation on NiO. If the Ni^{3+}-$(OH)_{ads}$-induced C-C bond cleavage does not work, the final electrooxidation product of CH_2OH-CH_2OH is COOH-COOH. The possible electrooxidation products of CH_2OH-CH_2OH are COOH-COOH, HCOOH, and H_2CO_3 [18]. H_2CO_3 is a stable oxidation product. It is doubtful whether COOH-COOH or HCOOH can be further electrochemically oxidized on the NiO electrode. The HCOOH electrooxidation on NiO is sluggish with a low reaction current. According to HPLC, the HCOOH containing nucleophilic aldehyde group was electrochemically oxidized to H_2CO_3 with a low Faraday efficiency. The deep oxidation of HCOOH to H_2CO_3 can influence the concentrations of HCOOH and H_2CO_3, so it is inappropriate to study reaction pathways based on the concentration of HCOOH or H_2CO_3. The anodic polarization curves of NiO electrode in 1 M KOH with/without 50 mM COOH-COOH are almost the same. The concentration of COOH-COOH remained unchanged during electrolysis, which proved that COOH-COOH could not be electrochemically oxidized on the NiO electrode.

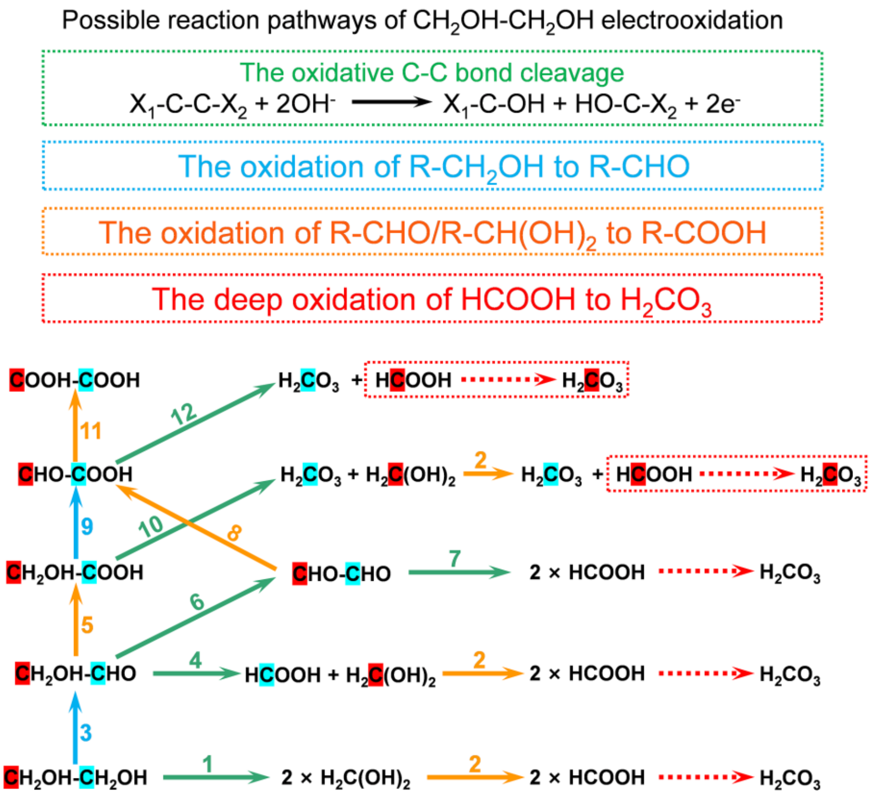

Figure S38. Schematic diagram showing possible reaction pathways of the electrooxidation of CH_2OH-CH_2OH.

The CH_2OH-CH_2OH electrooxidation pathway is composed of multiple basic electrochemical/non-electrochemical steps. Four basic reaction processes were adopted to describe different possible reaction pathways: (1) The oxidative C-C bond cleavage of X_1-C-C-X_2 to X_1-C-OH and X_2-C-OH; (2) The electrooxidation of R-CH_2OH to R-CHO; (3) The electrooxidation of R-CHO/R-$CH(OH)_2$ to R-COOH; (4) The sluggish deep electrooxidation of HCOOH to H_2CO_3. There are seven possible pathways for the electrooxidation of CH_2OH-CH_2OH, including twelve different basic processes. For the electrooxidation of CH_2OH-CH_2OH to COOH-COOH, there are two electrooxidation pathways (without the oxidative C-C bond cleavage): (1) Process 3, Process 5, Process 9, and Process 11; (2) Process 3, Process 6, Process 8, and Process 11. For the electrooxidation of CH_2OH-CH_2OH to HCOOH (two molecules), there are three electrooxidation pathways: (1) Process 1 and Process 2; (2) Process 3, Process 4, and Process 2; (3) Process 3, Process 6, and Process 7. Given the high selectivity of HCOOH (~95%) for the CH_2OH-CH_2OH electrooxidation on the NiO electrode, the possible reaction intermediates are $H_2C(OH)_2$, CH_2OH-CHO, and CHO-CHO.

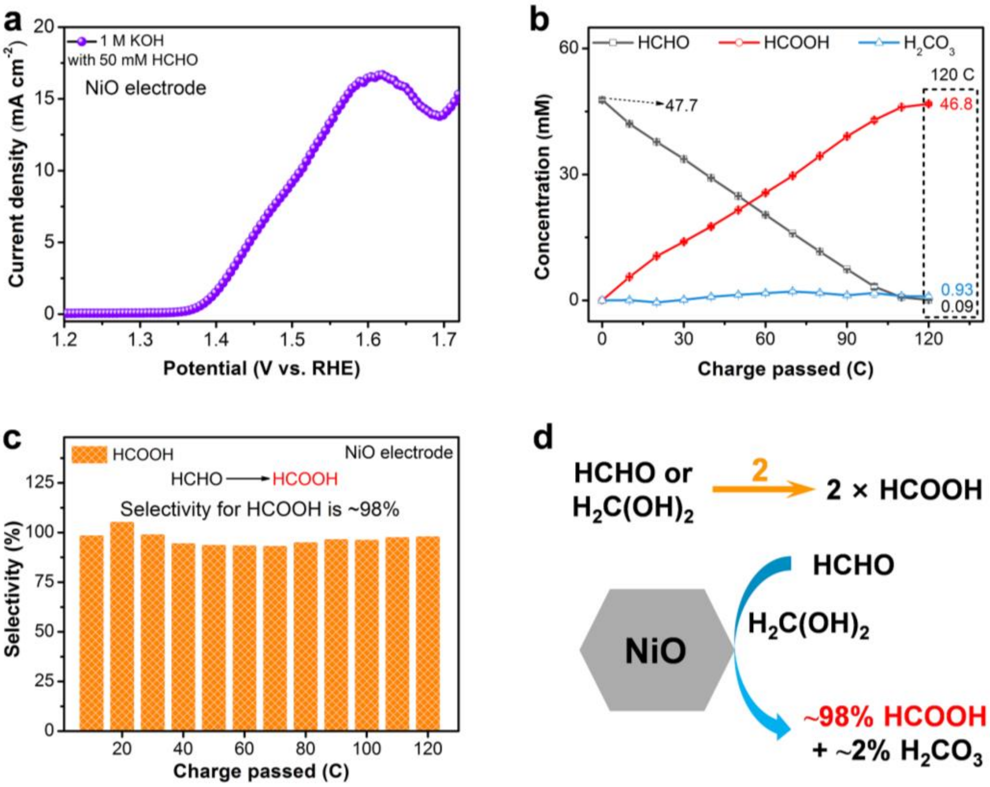

Figure S39. (a) Anodic polarization curve of NiO electrode in 1 M KOH with 50 mM HCHO. (b) Concentration changes of HCHO and its oxidation products (HCOOH and H_2CO_3) versus charge passed during the electrooxidation of HCHO on NiO. (c) Selectivity of HCOOH versus charge passed for the electrooxidation of HCHO on NiO. (d) Schematic diagram showing the electrooxidation of HCHO on NiO.

Because of the reversible hydration of HCHO to $H_2C(OH)_2$, the electrooxidation of HCHO on NiO electrode was investigated to analyze the Process 2 (the electrooxidation of $H_2C(OH)_2$ to HCOOH). NiO has an excellent electrochemical performance for the electrooxidation of HCHO/$H_2C(OH)_2$. For the HCHO electrooxidation on NiO, HCHO can be electrochemically oxidized to HCOOH with a high Faraday efficiency (above 90%). The selectivity of HCOOH is ~ 98%, and ~ 2% H_2CO_3 is generated due to the sluggish deep electrooxidation of HCOOH to H_2CO_3.

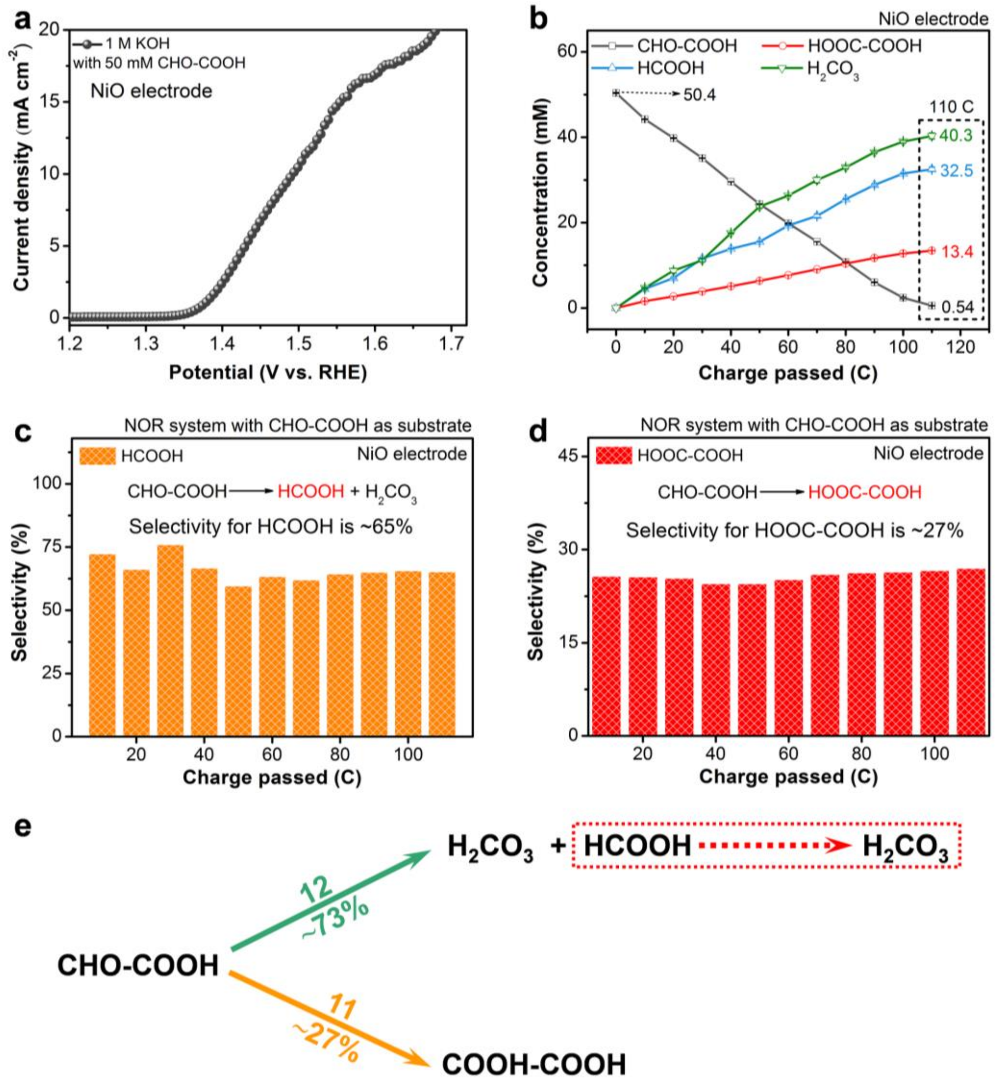

Figure S40. (a) Anodic polarization curve of NiO electrode in 1 M KOH with 50 mM CHO-COOH. (b) Concentration changes of CHO-COOH and its oxidation products (HOOC-COOH, HCOOH, and H_2CO_3) versus charge passed during the electrooxidation of CHO-COOH on NiO. (c, d) Selectivities of HCOOH (c) and HOOC-COOH (d) versus charge passed for the electrooxidation of CHO-COOH on NiO. (e) Schematic diagram showing reaction pathway of the CHO-COOH electrooxidation on NiO.

For the CHO-COOH electrooxidation on NiO, there are two different possible reaction pathways: (1) CHO-COOH is electrochemically oxidized to HCOOH and H_2CO_3 due to the oxidative C-C bond cleavage (Process 12); (2) CHO-COOH is electrochemically oxidized to COOH-COOH (Process 11). After the CHO-COOH (the concentration: 50.4 mM) electrooxidation on NiO, the concentrations of CHO-COOH, HCOOH, H_2CO_3, and COOH-COOH are 0.54, 32.5, 40.3, and 13.4 mM, respectively. The selectivities of HCOOH and COOH-COOH are ~65% and ~27%, respectively. Hence, for the CHO-COOH electrooxidation on NiO, the probabilities of Process 12 and Process 11 are ~73% and ~27%, respectively. The sluggish deep electrooxidation of HCOOH to H_2CO_3 leads to the lower selectivity of HCOOH (~65%) than the theoretical value (~73%).

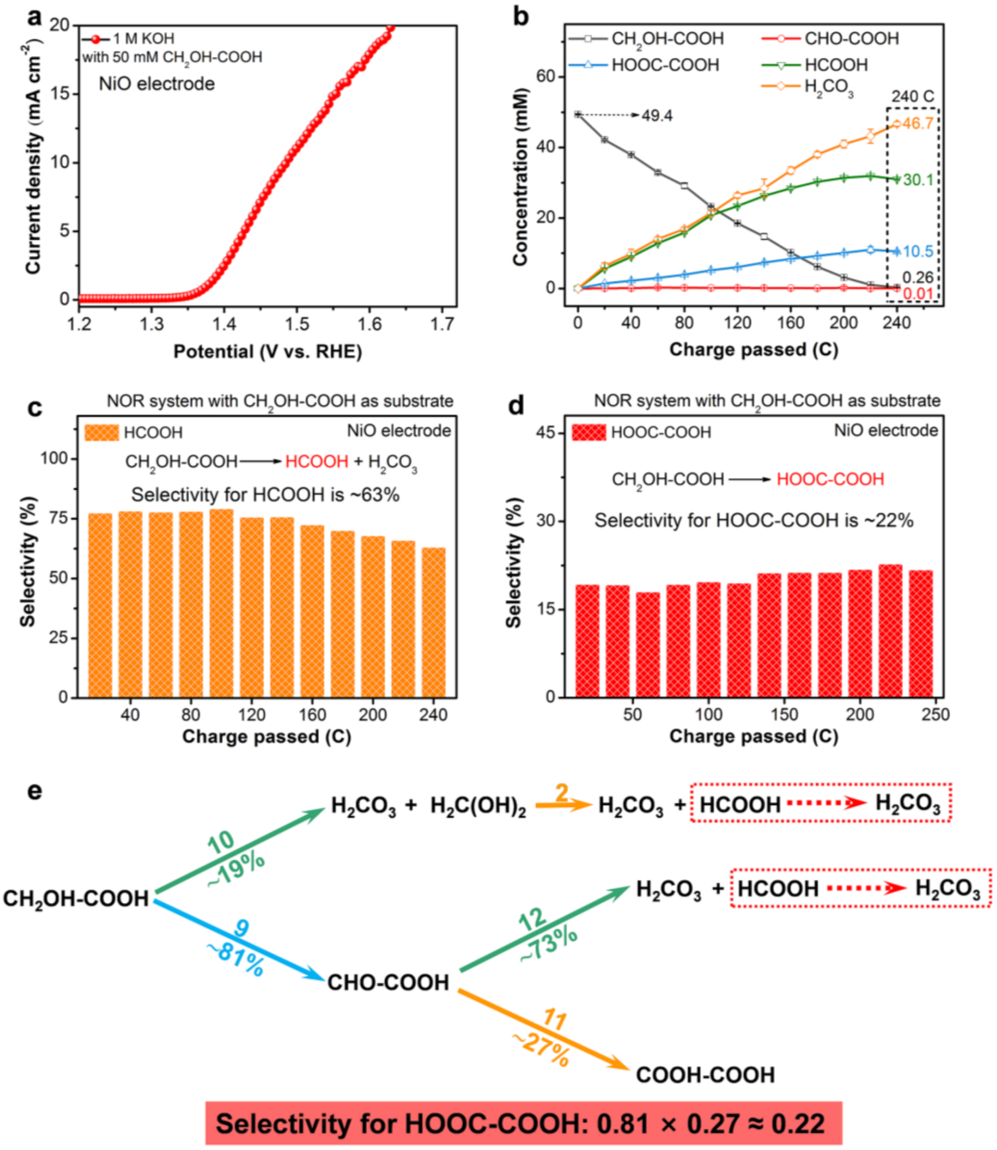

Figure S41. (a) Anodic polarization curve of NiO electrode in 1 M KOH with 50 mM CH_2OH-COOH. (b) Concentration changes of CH_2OH-COOH and its oxidation products (CHO-COOH, HOOC-COOH, HCOOH, and H_2CO_3) versus charge passed during the electrooxidation of CH_2OH-COOH on NiO. (c, d) Selectivities of HCOOH (c) and HOOC-COOH (d) versus charge passed for the electrooxidation of CH_2OH-COOH on NiO. (e) Schematic diagram showing reaction pathways of the CH_2OH-COOH electrooxidation on NiO.

For the CH_2OH-COOH electrooxidation on NiO, there are two different possible reaction processes at the first stage: (1) CH_2OH-COOH is electrochemically oxidized to $H_2C(OH)_2$ and H_2CO_3 (Process 10); (2) CH_2OH-COOH is electrochemically oxidized to CHO-COOH (Process 9). For the electrooxidation of CH_2OH-COOH on NiO, the selectivities of HCOOH and COOH-COOH are ~63% and ~22%, respectively. The electrooxidation of CH_2OH-COOH to COOH-COOH is composed of the first Process 9 and the subsequent Process 11. In theory, the selectivity of COOH-COOH (~22%) is the product of the probability of Process 9 and the probability of Process 11 (~27%). Hence, the probabilities of Process 9 and Process 10 are ~81% and ~19%, respectively.

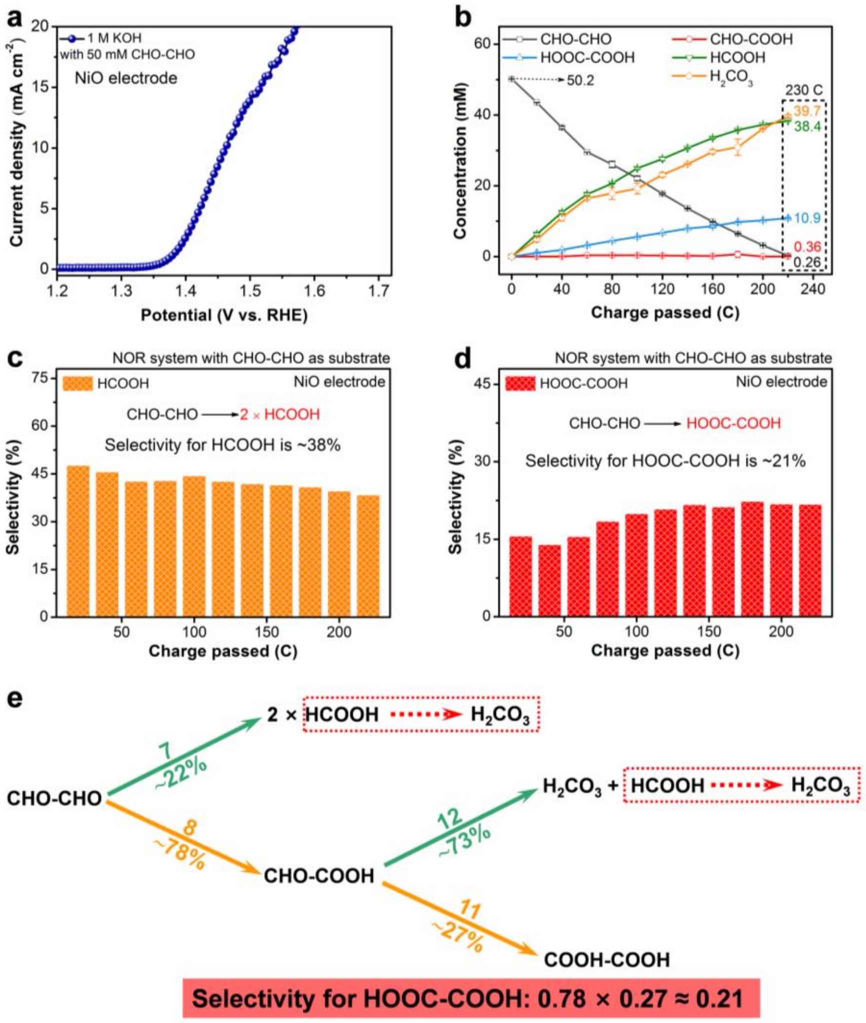

Figure S42. (a) Anodic polarization curve of NiO electrode in 1 M KOH with 50 mM CHO-CHO. (b) Concentration changes of CHO-CHO and its oxidation products (CHO-COOH, HOOC-COOH, HCOOH, and H_2CO_3) versus charge passed during the electrooxidation of CHO-CHO on NiO. (c, d) Selectivities of HCOOH (c) and HOOC-COOH (d) versus charge passed for the electrooxidation of CHO-CHO on NiO. (e) Schematic diagram showing reaction pathways of the CHO-CHO electrooxidation on NiO.

For the electrooxidation of CHO-CHO on NiO, there are two different possible reaction processes at the first stage: (1) CHO-CHO is electrochemically oxidized to two HCOOH molecules (Process 7); (2) CHO-CHO is electrochemically oxidized to CHO-COOH (Process 8). For the electrooxidation of CHO-CHO on NiO, the selectivities of HCOOH and COOH-COOH are ~38% and ~21%, respectively. The electrooxidation of CHO-CHO to COOH-COOH is composed of the first Process 8 and the subsequent Process 11. In theory, the selectivity of COOH-COOH (~21%) is the product of the probability of Process 8 and the probability of Process 11 (~27%). Hence, for the electrooxidation of CHO-CHO on NiO, the probabilities of Process 8 and Process 7 are ~78% and ~22%, respectively.

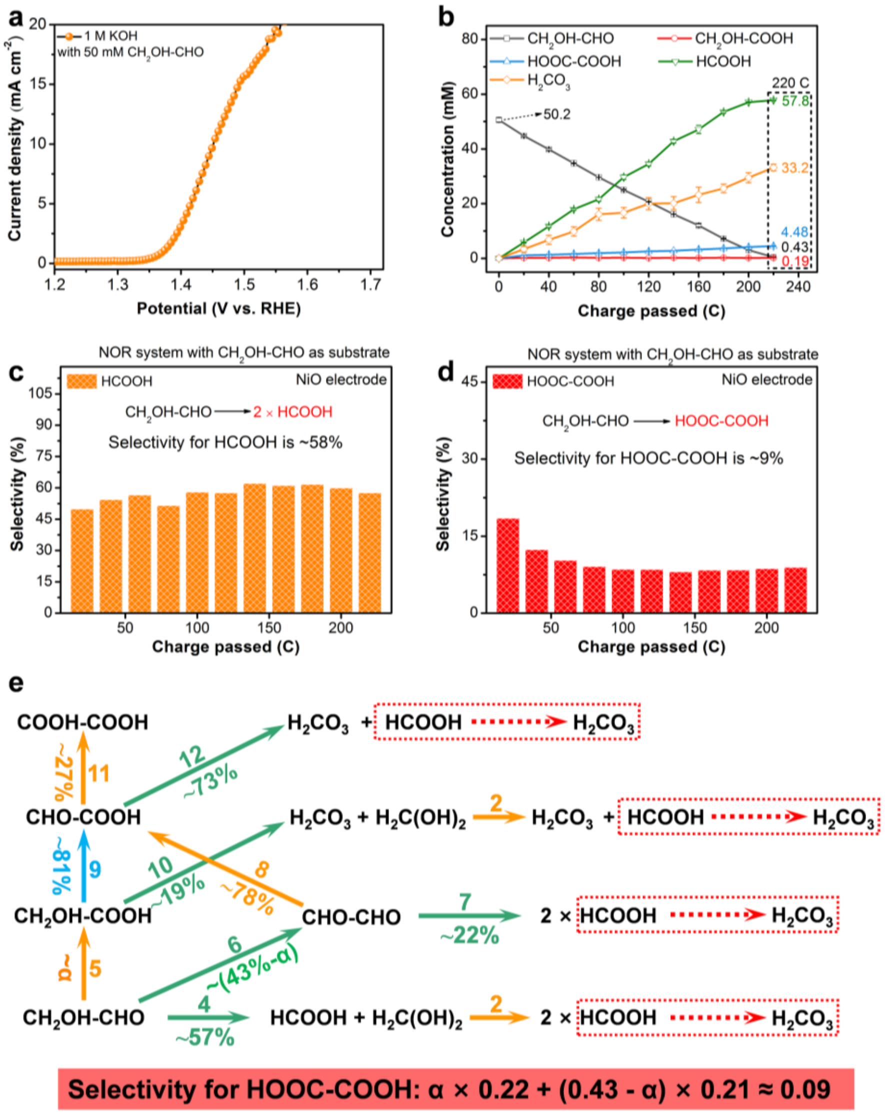

Figure S43. (a) Anodic polarization curve of NiO electrode in 1 M KOH with 50 mM CH_2OH-CHO. (b) Concentration changes of CH_2OH-CHO and its oxidation products (CH_2OH-COOH, HOOC-COOH, HCOOH, and H_2CO_3) versus charge passed during the electrooxidation of CH_2OH-CHO on NiO. (c, d) Selectivities of HCOOH (c) and HOOC-COOH (d) versus charge passed for the electrooxidation of CH_2OH-CHO on NiO. (e) Schematic diagram showing reaction pathways of the CH_2OH-CHO electrooxidation on NiO.

For the electrooxidation of CH_2OH-CHO on NiO, there are three different possible processes at the first stage: (1) CH_2OH-CHO is electrochemically oxidized to $H_2C(OH)_2$ and HCOOH (Process 4); (2) CH_2OH-CHO is electrochemically oxidized to CH_2OH-COOH (Process 5); (3) CHO-CH_2OH is electrochemically oxidized to CHO-CHO (Process 6). For the electrooxidation of CH_2OH-CHO on NiO, the selectivities of HCOOH and COOH-COOH are ~58% and ~9%, respectively. For the electrooxidation of CHO-CHO or CH_2OH-COOH, the selectivity of COOH-COOH is ~21-22%. Hence, for the electrooxidation of CH_2OH-CHO on NiO, the probability of Process 4 is ~57%, and the probabilities of Process 5 and Process 6 add up to ~43%. It is generally assumed that the electrooxidation of aldehyde group (Process 5) takes precedence over the electrooxidation of hydroxy group (Process 6) [19].

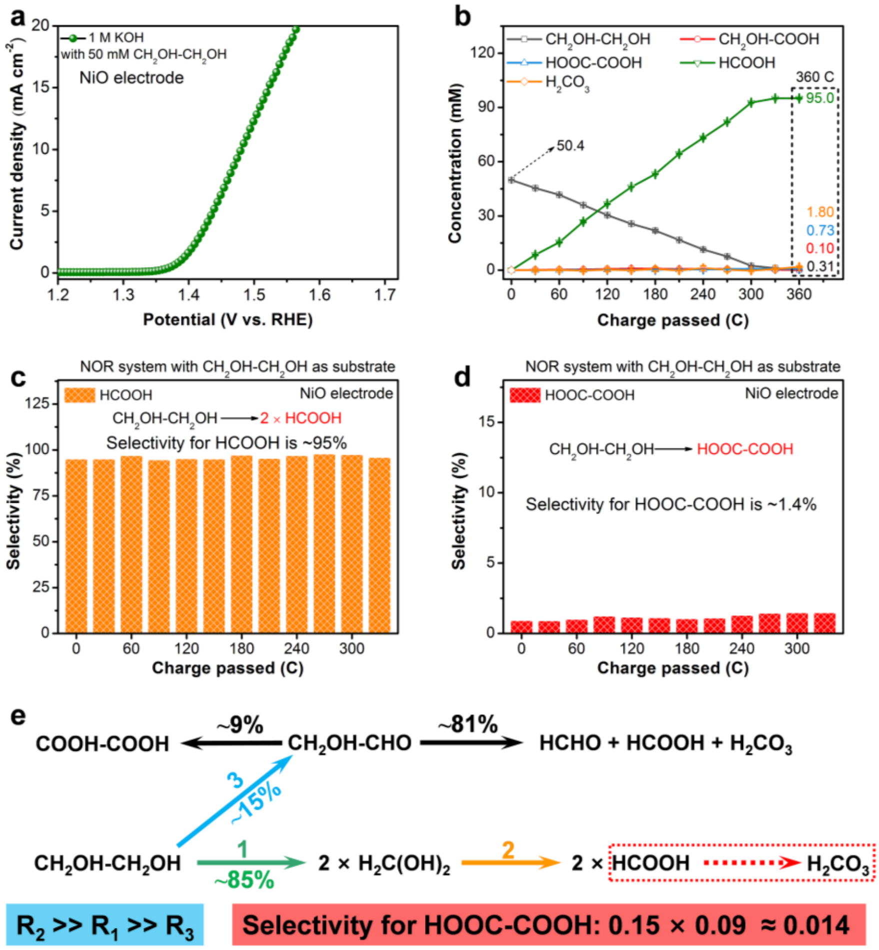

Figure S44. (a) Anodic polarization curve of NiO electrode in 1 M KOH with 50 mM CH_2OH-CH_2OH. (b) Concentration changes of CH_2OH-CH_2OH and its oxidation products (CH_2OH-COOH, HOOC-COOH, HCOOH, and H_2CO_3) versus charge passed during the electrooxidation of CH_2OH-CH_2OH on NiO. (c, d) Selectivities of HCOOH (c) and HOOC-COOH (d) versus charge passed for the electrooxidation of CH_2OH-CH_2OH on NiO. (e) Schematic diagram showing reaction pathways of the CH_2OH-CH_2OH electrooxidation on NiO.

For the CH_2OH-CH_2OH electrooxidation on NiO, there are two different possible processes at the first stage: (1) CH_2OH-CH_2OH is electrochemically oxidized to $H_2C(OH)_2$ (Process 1); (2) CH_2OH-CH_2OH is electrochemically oxidized to CH_2OH-CHO (Process 2). For the electrooxidation of CH_2OH-CH_2OH on NiO, the selectivities of HCOOH and COOH-COOH are ~95% and ~1.4%, respectively. In theory, the selectivity for the electrooxidation of CH_2OH-CH_2OH to COOH-COOH (~1.4%) is the product of the probability of Process 2 (the electrooxidation of CH_2OH-CH_2OH to CH_2OH-CHO) and the selectivity for the electrooxidation of CH_2OH-CHO to COOH-COOH (~9%). Hence, for the electrooxidation of CH_2OH-CH_2OH on NiO, the probabilities of Process 1 and Process 3 are ~85% and ~15%, respectively. To sum up, the main reaction pathway for the electrooxidation of CH_2OH-CH_2OH on NiO consists of two stages: (1) Firstly, CH_2OH-CH_2OH is electrochemically oxidized to two $H_2C(OH)_2$ molecules (Process 1) due to EOM involving C-C bond cleavage; (2) Second, $H_2C(OH)_2$ is electrochemically oxidized to HCOOH (Process 2) due to EOM involving HAT.

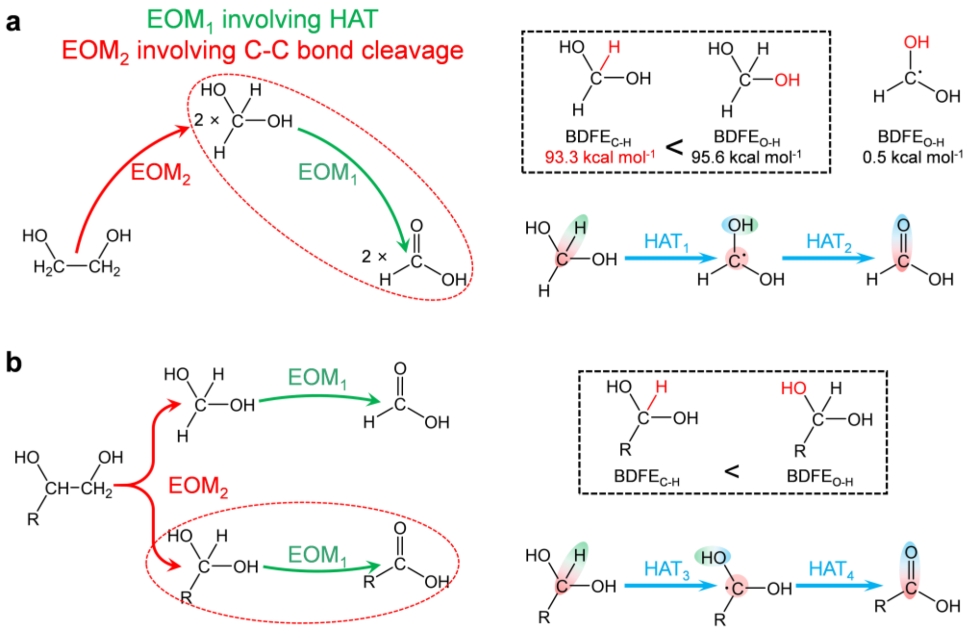

Figure S45. Reaction pathways for the electrooxidations of CH_2OH-CH_2OH (a) and R-CHOH-CH_2OH (b) on NiO. The EOM involving C-C bond cleavage preferentially occurs during the CH_2OH-CH_2OH electrooxidation on NiO, and the product is $H_2C(OH)_2$.

For $H_2C(OH)_2$, BDFEs of the C-H bond and the O-H bond are 93.3 and 95.6 kcal mol^{-1}, respectively. Hence, for the oxidation of $H_2C(OH)_2$ to HCOOH, the first HAT step (HAT_1) is the dehydrogenation of $H_2C(OH)_2$ to H·C$(OH)_2$, and the second step (HAT_2) is the dehydrogenation of H·C$(OH)_2$ to HCOOH. R-CHOH-CH_2OH can be electrochemically oxidized to R-COOH and HCOOH. Depending on the reaction pathway for the CH_2OH-CH_2OH electrooxidation, we can make a reasonable assumption that the C-C bond cleavage of R-CHOH-CH_2OH to R-CH$(OH)_2$ and $H_2C(OH)_2$ preferentially occurs during the electrooxidation of R-CHOH-CH_2OH on NiO. Generally speaking, the BDFE of alpha-C-H bond in R-CH$(OH)_2$ is less than the BDFE of O-H bond in R-CH$(OH)_2$. Hence, the dehydrogenation of alpha-C-H bond in R-CH$(OH)_2$ (HAT_3) takes precedence over the dehydrogenation of O-H bond in R-CH$(OH)_2$ (HAT_4).

Table S1. Optimum fit parameters for the impedance date of NiO electrode in OER system (1 M KOH).

E V vs. RHE	R_s Ω	CPE_1-Yo μS s^n	CPE_1-n	R_1 Ω	C_1 μF	τ_1 s	CPE_2-Yo μS s^n	CPE_2-n	R_2 Ω	C_2 μF	τ_2 s
1.10	4.93	44.78	0.91	2600.00	36.22	0.0942					
1.20	4.88	50.36	0.89	2088.00	38.56	0.0805					
1.25	4.84	54.87	0.88	1692.00	40.18	0.0680					
1.30	4.84	65.74	0.86	1468.00	45.16	0.0663					
1.35	4.88	67.45	0.87	722.70	42.30	0.0306					
1.40	4.74	147.90	0.81	525.90	81.21	0.0427					
1.45	4.68	183.60	0.81	362.80	97.86	0.0355					
1.50	4.64	167.30	0.81	244.70	77.79	0.0190					
1.55	4.72	159.70	0.85	62.65	71.90	0.0045	467.30	0.81	3184.00	512.33	1.6313
1.60	4.76	118.60	0.90	34.68	63.07	0.0022	529.60	0.76	390.50	326.10	0.1273
1.65	5.19	110.20	0.90	24.00	59.06	0.0014	557.70	0.75	176.40	260.22	0.0459
1.70	5.61	119.60	0.88	20.48	52.73	0.0011	460.50	0.79	106.40	203.18	0.0216

Table S2. Optimum fit parameters for the impedance date of NiO electrode in NOR system (1 M KOH with 0.5 M ethanol).

E V vs. RHE	R_s Ω	CPE_1-Yo μS s^n	CPE_1-n	R_1 Ω	C_1 μF	τ_1 s	CPE_2-Yo μS s^n	CPE_2-n	R_2 Ω	C_2 μF	τ_2 s
1.10	4.31	92.92	0.71	1653.00	43.57	0.07202					
1.20	4.03	153.60	0.66	1462.00	71.01	0.10382					
1.25	4.27	117.40	0.69	496.20	33.50	0.01662					
1.30	4.22	138.40	0.68	370.70	33.90	0.01257					
1.35	4.71	62.07	0.79	82.70	14.85	0.00123	414.70	0.75	1318.00	339.25	0.4471
1.40	4.81	44.60	0.82	49.64	11.81	0.00059	454.50	0.73	252.20	206.31	0.0520
1.45	4.92	30.96	0.86	32.09	10.23	0.00033	484.10	0.70	122.80	146.74	0.0180
1.50	4.92	25.95	0.87	26.58	9.01	0.00024	529.40	0.71	82.61	143.00	0.0118
1.55	4.95	25.44	0.87	24.77	8.17	0.00020	594.60	0.73	61.50	172.62	0.0106
1.60	4.97	26.14	0.86	23.62	7.72	0.00018	609.80	0.76	52.32	207.79	0.0109
1.65	4.99	26.04	0.86	23.18	7.58	0.00018	610.40	0.78	46.42	229.60	0.0107
1.70	5.05	24.65	0.86	21.62	7.50	0.00016	655.30	0.78	44.05	246.89	0.0109

Table S3. Optimum fit parameters for the impedance date for the electrochemical oxidation-induced C-C bond cleavage on NiO electrode (1 M KOH with 0.5 M CH_3-CO-COOH).

E V vs. RHE	R_s Ω	CPE_1-Yo μS s^n	CPE_1-n	R_1 Ω	C_1 μF	τ_1 s	CPE_2-Yo μS s^n	CPE_2-n	R_2 Ω	C_2 μF	τ_2 s
1.10	5.057	45.42	0.7975	425.4	16.67	0.00709					
1.20	5.042	49.48	0.7914	271.9	15.89	0.00432					
1.25	5.037	51.54	0.7885	224.1	15.58	0.00349					
1.30	5.171	49.06	0.8176	112.8	15.39	0.00174					
1.35	5.22	44.84	0.8314	87.6	14.58	0.00128	368	0.7506	651.3	228.94	0.1491
1.40	5.268	40.4	0.8555	61.04	14.65	0.00089	353.9	0.7294	238.7	141.48	0.0338
1.45	5.269	36.45	0.8789	43.84	15.01	0.00066	298.8	0.7257	134.9	88.77	0.0120
1.50	5.327	30.69	0.9083	32.09	15.26	0.00049	245.8	0.728	102.3	62.08	0.0064
1.55	5.346	26.41	0.9304	24.27	15.24	0.00037	218.4	0.7312	85.21	50.49	0.0043
1.60	5.356	23.76	0.9445	19.86	15.15	0.00030	220.7	0.7269	75.79	47.46	0.0036
1.65	5.429	23.01	0.9429	18.14	14.36	0.00026	228.6	0.7318	68.51	49.83	0.0034
1.70	5.429	22.8	0.9437	16.84	14.26	0.00024	269.7	0.7202	66.07	56.41	0.0037

References

1 Kresse G and Hafner J. Ab initio molecular dynamics for open-shell transition metals. *Phys Rev B* 1993; **48**: 13115.

2 Kresse G and Furthmüller J. Efficient iterative schemes for ab initio total-energy calculations using a plane-wave basis set. *Phys Rev B* 1996; **54**: 11169.

3 Perdew JP, Burke K and Ernzerhof M. Generalized gradient approximation made simple. *Phys Rev Lett* 1996; **77**: 3865.

4 Zhao W, Bajdich M and Carey S *et al.* Water dissociative adsorption on NiO(111): energetics and structure of the hydroxylated surface. *ACS Catal* 2016; **6**: 7377-7384.

5 Lyons MEG, Doyle RL and Brandon MP. Redox switching and oxygen evolution at oxidized metal and metal oxide electrodes: iron in base. *Phys Chem Chem Phys* 2011; **13**: 21530-21551.

6 Lyons MEG and Brandon MP. Redox switching and oxygen evolution electrocatalysis in polymeric iron oxyhydroxide films. *Phys Chem Chem Phys* 2009; **11**: 2203-2217.

7 Song J, Wei C and Huang ZF *et al.* A review on fundamentals for designing oxygen evolution electrocatalysts. *Chem Soc Rev* 2020; **49**: 2196-2214.

8 Tao HB, Xu Y and Huang X *et al.* A general method to probe oxygen evolution intermediates at operating conditions. *Joule* 2019; **3**: 1498-1509.

9 Bai LC, Hsu CS and Alexander DTL *et al.* Double-atom catalysts as a molecular platform for heterogeneous oxygen evolution electrocatalysis. *Nat Energy* 2021; **6**: 1054-1066.

10 Chen A and Lipkowski J. Electrochemical and spectroscopic studies of hydroxide adsorption at the Au(111) electrode. *J Phys Chem B* 1999; **103**: 682-691.

11 Ramaswamy N. and Mukerjee S. Fundamental mechanistic understanding of electrocatalysis of oxygen reduction on Pt and non-Pt surfaces: acid versus alkaline media. *Adv Phys Chem* 2012; **2012**: 491604.

12 Deng YJ, Arenz M and Wiberg GKH. Equilibrium coverage of OH_{ad} in correlation with platinum catalyzed fuel cell reactions in $HClO_4$. *Electrochem Commun* 2015; **53**: 41-44.

13 Darcy JW, Koronkiewicz B, Parada GA *et al.* A continuum of proton-coupled electron transfer reactivity. *Acc Chem Res* 2018; **51**: 2391-2399.

14 Mayer JM. Understanding hydrogen atom transfer: from bond strengths to marcus theory. *Acc Chem Res* 2011; **44**: 36-46.

15 Shi SH, Liang YJ and Jiao N. Electrochemical oxidation induced selective C-C bond cleavage. *Chem Rev* 2021; **121**: 485-505.

16 Gupta KS and Sarkar T. Kinetics of the chromic acid oxidation of glyoxylic and pyruvic acids. *Tetrahedron* 1975; **31**: 123-127.

17 Lipmann F. A phosphorylated oxidation product of pyruvic acid. *J Biol Chem* 1940; **134**: 463-464.

18 Matsumoto T, Sadakiyo M and Ooi ML *et al.* CO_2-free power generation on an iron group nanoalloy catalyst via selective oxidation of ethylene glycol to oxalic acid in alkaline media. *Sci Rep* 2014; **4**: 5620.

19 Zhang N, Zou Y and Tao L, *et al.* Electrochemical oxidation of 5-hydroxymethylfurfural on nickel nitride/carbon nanosheets: reaction pathway determined by in situ sum frequency generation vibrational spectroscopy. *Angew Chem Int Edit* 2019; **58**: 15895-15903.
